# Crash testing machine learning force fields for molecules, materials, and interfaces: model analysis in the TEA Challenge 2023†

**DOI:** 10.1039/d4sc06529h

**Published:** 2025-02-10

**Authors:** Igor Poltavsky, Anton Charkin-Gorbulin, Mirela Puleva, Grégory Fonseca, Ilyes Batatia, Nicholas J. Browning, Stefan Chmiela, Mengnan Cui, J. Thorben Frank, Stefan Heinen, Bing Huang, Silvan Käser, Adil Kabylda, Danish Khan, Carolin Müller, Alastair J. A. Price, Kai Riedmiller, Kai Töpfer, Tsz Wai Ko, Markus Meuwly, Matthias Rupp, Gábor Csányi, O. Anatole von Lilienfeld, Johannes T. Margraf, Klaus-Robert Müller, Alexandre Tkatchenko

**Affiliations:** a Department of Physics and Materials Science, University of Luxembourg L-1511 Luxembourg Luxembourg alexandre.tkatchenko@uni.lu igor.poltavskyi@uni.lu; b Laboratory for Chemistry of Novel Materials, University of Mons B-7000 Mons Belgium; c Institute for Advanced Studies, University of Luxembourg Campus Belval L-4365 Esch-sur-Alzette Luxembourg; d Department of Engineering, University of Cambridge Trumpington Street Cambridge CB2 1PZ UK; e Swiss National Supercomputing Centre (CSCS) 6900 Lugano Switzerland; f Machine Learning Group, Technical University Berlin Berlin Germany; g BIFOLD, Berlin Institute for the Foundations of Learning and Data Berlin Germany; h Fritz-Haber-Institut der Max-Planck-Gesellschaft Berlin Germany; i Vector Institute for Artificial Intelligence Toronto ON M5S 1M1 Canada; j Wuhan University, Department of Chemistry and Molecular Sciences 430072 Wuhan China; k Department of Chemistry, University of Basel Klingelbergstrasse 80 CH-4056 Basel Switzerland; l Chemical Physics Theory Group, Department of Chemistry, University of Toronto St George Campus Toronto ON Canada; m Friedrich-Alexander-Universität Erlangen-Nürnberg, Computer-Chemistry-Center Nägelsbachstraße 25 91052 Erlangen Germany; n Department of Chemistry, University of Toronto St George Campus Toronto ON Canada; o Acceleration Consortium, University of Toronto 80 St George St Toronto ON M5S 3H6 Canada; p Heidelberg Institute for Theoretical Studies Heidelberg Germany; q Department of NanoEngineering, University of California San Diego 9500 Gilman Dr, Mail Code 0448 La Jolla CA 92093-0448 USA; r Luxembourg Institute of Science and Technology (LIST) L-4362 Esch-sur-Alzette Luxembourg; s Department of Materials Science and Engineering, University of Toronto St George Campus Toronto ON Canada; t Department of Physics, University of Toronto St George Campus Toronto ON Canada; u University of Bayreuth, Bavarian Center for Battery Technology (BayBatt) Bayreuth Germany; v Department of Artificial Intelligence, Korea University Seoul South Korea; w Max Planck Institut für Informatik Saarbrücken Germany; x Google DeepMind Berlin Germany

## Abstract

Atomistic simulations are routinely employed in academia and industry to study the behavior of molecules, materials, and their interfaces. Central to these simulations are force fields (FFs), whose development is challenged by intricate interatomic interactions at different spatio-temporal scales and the vast expanse of chemical space. Machine learning (ML) FFs, trained on quantum-mechanical energies and forces, have shown the capacity to achieve sub-kcal (mol^−1^ Å^−1^) accuracy while maintaining computational efficiency. The TEA Challenge 2023 rigorously evaluated commonly used MLFFs across diverse applications, highlighting their strengths and weaknesses. Participants trained their models using provided datasets, and the results were systematically analyzed to assess the ability of MLFFs to reproduce potential energy surfaces, handle incomplete reference data, manage multi-component systems, and model complex periodic structures. This publication describes the datasets, outlines the proposed challenges, and presents a detailed analysis of the accuracy, stability, and efficiency of the MACE, SO3krates, sGDML, SOAP/GAP, and FCHL19* architectures in molecular dynamics simulations. The models represent the MLFF developers who participated in the TEA Challenge 2023. All results presented correspond to the state of the ML architectures as of October 2023. A comprehensive analysis of the molecular dynamics results obtained with different MLFFs will be presented in the second part of this manuscript.

## Abstract 2

Assessing the performance of modern machine learning force fields across diverse chemical systems to identify their strengths and limitations within the TEA Challenge 2023.

## Graphical abstract

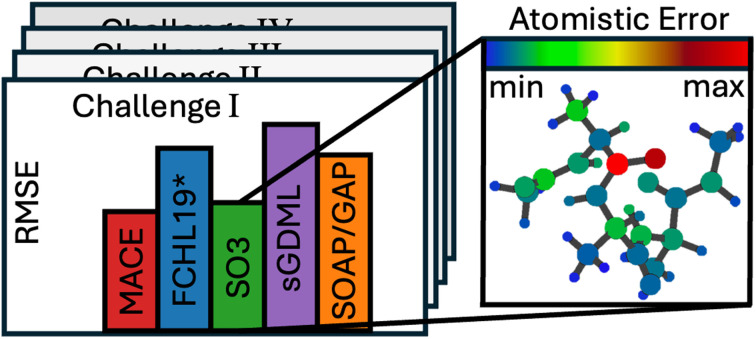

## 1. Introduction

Robust and accurate computer simulations of chemical systems are essential for multiple applications ranging from biomedical research to the development of semiconductors and solar cells. Central to these simulations are force fields (FFs), which play a pivotal role in determining their accuracy and reliability. The complexity of intra- and intermolecular interactions within realistic systems, combined with the vastness of chemical compound space, makes developing effective FFs a formidable challenge. Despite the long-standing use of FFs, only recently has the machine learning (ML) paradigm enabled FFs to achieve quantum-chemical accuracy for broad chemical spaces while maintaining computational efficiency. In less than two decades, ML-based force fields (MLFFs) have evolved significantly. They have progressed from achieving qualitatively correct results for relatively simple periodic systems^1–10^ and small molecules^11–22^ to simulating nanoseconds of dynamics under realistic conditions for molecules and materials containing thousands to millions of atoms, all while maintaining root mean squared errors in forces within a fraction of one kcal (mol^−1^ Å^−1^)^23–32^ compared to the forces produced with quantum chemistry reference methods (at least for small fragments).

Despite their remarkable success in the world of hard materials, for organic chemistry and softer materials, developing generally useful MLFFs remains an ongoing challenge. Specifically, issues such as reliable transferability (in chemical space), reactivity and scalability (in terms of system size) have yet to be fully satisfactorily addressed.^33–44^ Along the road of progress for MLFFs, a crucial role has been played by the significant efforts that have been dedicated to assessing the state of the field and identifying areas for improvement.^45–62^ One of the main challenges here is that due to the increasing complexity of the systems, MLFFs require specialized expertise to identify the correct performance metrics, making reliable error estimation difficult. A significant risk can occur when MLFFs do not fail outright but produce seemingly reasonable yet ultimately incorrect results. Identifying such cases in large and complex systems requires deeper assessment than mere comparison to reference *ab initio* calculations, as those calculations can be computationally prohibitive or even unfeasible.

An alternative approach to assessing models in areas of chemical space unreachable by traditional quantum methods is to reproduce the same computer simulations using different MLFFs. A consistent outcome of those simulations, especially by different architectures, would indicate a high likelihood of a correct result. However, when discrepancies arise, this is indicative of possible mistakes in one or all MLFF results, and the simulation results should be interrogated further. Therefore, performing a comprehensive comparative analysis of the performance of various MLFF models across different types of systems is crucial for understanding their actual application ranges. Similar work has been done on reproducibility for Density Functional Theory (DFT) codes,^63,64^ which has led to increasing trust in the DFT calculations, and those are now frequently used as a reference in MLFF tests whenever possible.

**Fig. 1:**
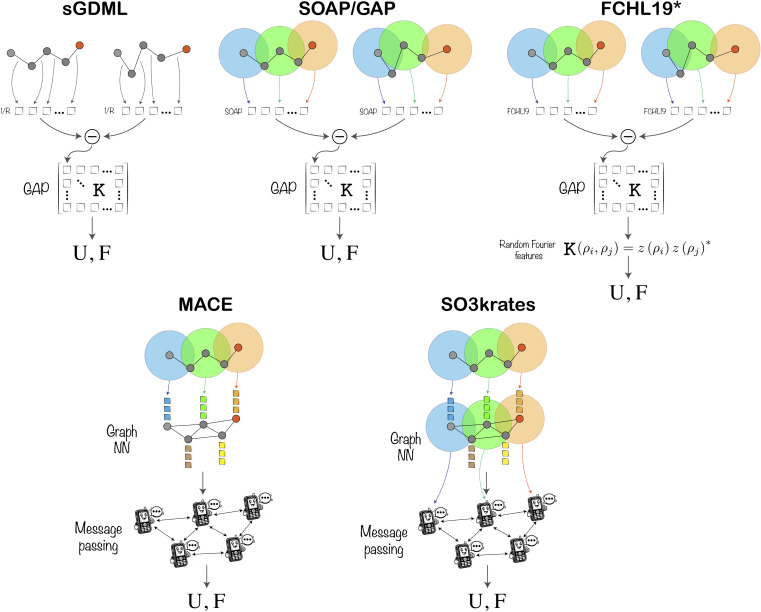
Schematic representation of the ML architectures involved in the TEA Challenge 2023, in which U and F are the output energies and forces, respectively. Further details of the methods and their implementation are available in the ESI.†

The “Crash testing machine learning force fields for molecules, materials, and interfaces” *i.e.* the TEA Challenge aims to provide a platform for rigorous testing of commonly used MLFFs across diverse applications. The TEA Challenge 2023 commenced with a workshop, which gathered MLFF developers. While many notable MLFF developers of recent years were invited, the TEA Challenge 2023 as presented here only comprises those who undertook the challenge. The developers were provided with training datasets and limited information about the details of data generation in order to ensure that the data could not be extended unilaterally. They were tasked with training their models to the degree that they chose as best suiting the task and presenting their results; in particular, each developer made their own choices and trade-offs regarding model size, accuracy and computational efficiency. The TEA Challenge 2023 spans a range of models from lighter kernel regression models with 123 000 trainable parameters through lighter neural networks (NNs) with 487 613 to heavier neural networks with 2 983 184. The results were further analysed by the organisers, who were not involved in the models' training process. The models could be improved and resubmitted based solely on outcomes from predefined test sets presented during the workshop. A schematic representation of ML models submitted in the TEA Challenge 2023 is shown in Fig. 1, while their details are available in the ESI.† Subsequently, the organisers conducted tests of running molecular dynamics (MD) simulations using the final MLFF models under identical conditions within the same platform on the same High Performance Cluster (HPC). At this stage, the models could no longer be altered nor could developers influence the outcomes of the analysis. This approach simulates realistic application conditions for MLFFs, where the ground truth is unknown, highlighting potential issues practitioners might encounter. The selected systems for MDs were designed to be manageable yet challenging within most modern MLFF architectures, diverse, and unbiased towards any existing ML models.

This article is divided into five sections as follows. Section II introduces the four datasets used in each of the Challenges I−IV, while Section III details the challenges themselves. Section IV analyzes the accuracy of MLFFs on the test sets, their stability during MD simulations, and their efficiency based on the computational resources required to produce 1 million steps of a classical MD simulation (equivalent to 1 nanosecond of dynamics with 1 femtosecond time steps). Some pitfalls of the MLFF implementations are also discussed. Our findings are summarized in Section V. Additionally, a detailed description of the MLFFs that participated in the TEA Challenge 2023, along with results for PhysNet^65^ and a benchmark of hydrogen under pressure, can be found in the ESI.† A comprehensive analysis of the MD simulations within each of the four challenges is provided in the follow-up article, “Crash testing machine learning force fields for molecules, materials, and interfaces: molecular dynamics in the TEA Challenge 2023”.^66^

## 2. Datasets

The energies and forces for all the systems listed below were obtained using DFT with semi-local and hybrid exchange–correlation functionals and MBD/MBD-NL dispersion interactions within the FHI-aims code.^67–69^

(I) The Alanine tetrapeptide dataset (Ac-Ala3-NHMe, 42 atoms) is a part of the MD22 benchmark dataset.^70^ It was generated as a single NVT MD trajectory sampled at a temperature of 500 K with a time step of 1 fs. The corresponding potential energy and atomic forces are calculated with the Perdew–Burke–Ernzerhof (PBE) exchange–correlation functional^71^ and Many-Body Dispersion (MBD)^72,73^ method. The dataset contains a total of 85 109 structures.

(II) The *N*-acetylphenylalanyl-pentaalanyl-lysine dataset (Ac-Phe-Ala5-Lys, 100 atoms) was specifically generated for the TEA Challenge 2023. It contains a limited sampling near the 200 lowest energy conformers identified using the CREST software package.^74^ The sampling was performed by running NVT MD simulations at a temperature of 500 K with a time step of 0.5 fs starting from each conformer's equilibrium structure. All the trajectories are 250 fs long and contain 500 configurations. This gives a total of 100 000 reference structures with energies and forces computed using the PBE0 (ref. 75) exchange–correlation functional and nonlocal MBD (MBD-NL)^76^ method to describe dispersion interactions. The dataset contains the indices of the MD trajectories (starting conformers) from which the given geometry was extracted. The Ac-Phe-Ala5-Lys is protonated in all the calculations.

(III) The 1,8-Naphthyridine molecule adsorbed on the graphene dataset (C_8_H_6_N_2_/C_98_, 114 atoms) was specifically generated for the TEA Challenge 2023. It consists of six independent NVT MD runs performed at a temperature of 500 K with a time step of 1 fs. The dataset contains 15 000 reference structures with forces and energies computed at the PBE + MBD-NL level of theory.

(IV) The tetragonal phase Methylammonium Lead Iodide perovskite dataset (MAPbI_3_, 384 atoms) was created by extracting the train, validation, and test sets (selected based on the energy distribution using the procedure implemented within the sGDML software package^15,45^) from the MD trajectory published in ref. 77. For the TEA Challenge 2023, 698 structures were recomputed at the PBE + MBD-NL level of theory.

**Fig. 2:**
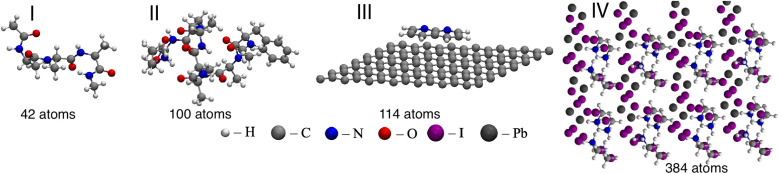
Snapshots of system geometries from: I – Ac-Ala3-NHMe, II – Ac-Phe-Ala5-Lys, III – C_8_H_6_N_2_/C_98_, and IV – MAPbI_3_ datasets.

Notably, only the Alanine tetrapeptide dataset was published before the TEA Challenge 2023. Therefore, the MLFF developers had limited information about three out of four datasets. Visualisations of the atomic structures are shown in Fig. 2 for the four datasets.

## 3. Challenges

The TEA Challenge 2023 consisted of four independent challenges testing the limits of modern MLFFs. All the TEA 2023 participants were provided with the same information, train, validation, and test datasets, which can be found in the Zenodo archive https://doi.org/10.5281/zenodo.14138387. None of them were involved in the TEA Challenge 2023 preparations, ensuring a level playing field. The participants presented their results in a dedicated workshop. After the workshop ended, participants were allowed to improve their models. The TEA 2023 team performed all the test simulations afterwards using the same HPC hardware and settings for all provided MLFF models to ensure a fair comparison. The MLFF performance tests (model evaluations per second) were done under the same conditions by running simulations using the same script for all MLFF models on dedicated HPC nodes, ensuring that the results were free from potential interference from other jobs.

The first challenge aims to verify the ability of MLFF models to reproduce the potential energy surface (PES) for flexible organic molecules, where the training dataset is chosen to include only folded or alternatively only unfolded configurations. We use the Alanine tetrapeptide dataset split into training, validation, and test sets using three different strategies to do so. As a benchmark, we start with MLFFs trained on representative samples of all possible extended and compact Ac-Ala3-NHMe structures with training/validation datasets of the sizes 200, 400, 600, 800, and 1000 to validate the convergence of the models. Next, we divide the entire Alanine tetrapeptide dataset into two subsets based on the distance between the farthest non-hydrogen atoms as a measure of the compactness of the molecule. 70% of the most compact structures form the folded dataset, while the remaining 30% form the unfolded one. The classification is based solely on whether the corresponding interatomic distance is larger or smaller than the empirically defined threshold of 10.06 Å found suitable for this dataset. The challenge posed to the participants was to train MLFFs using only one of the proposed subsets of configurations (provided to all participants) and to predict the unseen data from the other subset. The evaluation criteria are the accuracy and stability of three types of MLFF models. In this way, we test the ability of MLFFs to extrapolate in the configurational space. All the analyses for accuracy, stability, and performance of the provided MLFF models are done based on the most accurate models trained on 1000 reference geometries. Different training/validation test sizes were used solely to ensure the convergence of the ML models during the training process.

For the stability and performance tests, the trained models provided by participants were used by the TEA 2023 evaluation team to run 12 independent NVT MD simulations employing a Langevin thermostat with a friction coefficient of 10^−3^ fs^−1^ and a time step of 1 fs to measure the trajectory's average length before the system enters a nonphysical state (*i.e.* breaking chemical bonds). The 12 MD runs were started at a temperature of 300 K from 12 fixed nonidentical system configurations. The 12 starting configurations were always chosen from the training datasets. When all 12 trajectories reached 1 ns length without entering an unphysical state, the same models were tested at an increased temperature of 500 K. For the most robust models, 12 MDs were run once more at 700 K. The starting conditions and the MD settings were identical for all MLFFs participating in the challenge.

The second challenge verifies the ability of MLFFs to deal with a different type of reference data incompleteness. It is based on the Ac-Phe-Ala5-Lys dataset. As a baseline, we have “complete” ML models trained on a fixed number (10 and 20) of randomly selected molecule configurations from each of the 200 MD trajectories. Additionally, we trained ML models on a separate dataset to simulate “incomplete” training data by sampling a fixed number (16 and 32) of randomly selected configurations from 125 out of the 200 MD trajectories. The remaining 75 trajectories were excluded from training and used as an “unseen” set. The accuracy of the MLFFs was compared for complete and incomplete ML models tested on both seen and unseen test datasets. Note that the training and validation dataset sizes are 2000 and 4000 for both types of models, and the datasets are the same for all MLFF architectures. For the analysis, we use only the models trained on 4000 reference geometries. The stability and performance of the MLFFs were estimated in the same manner as for Challenge I.

The third challenge assesses the ability of MLFFs to deal with multi-component interfaces. It is based on a 1,8-Naphthyridine molecule adsorbed on a pristine graphene sheet. While the 1,8-Naphthyridine molecule and the graphene sheet are, by themselves, trivial challenges for modern MLFFs, their combination introduces a new element: molecule–surface interaction. This interaction is non-covalent and small in strength compared to interatomic forces within the subsystems. At the same time, molecule–surface interaction determines many effects of practical interest, such as surface friction or adsorption. The participants were provided with training and validation datasets of the sizes 200, 400, 600, 800, and 1000. The test dataset in all cases was all configurations left in the 1,8-Naphthyridine molecule adsorbed on a pristine graphene sheet dataset after extracting the training and validation subsets. The stability and performance of the MLFFs were estimated in the same manner as for the first challenge using the MLFFs trained on the largest training set (1000 configurations) with periodic boundary conditions in two dimensions. One key difference in the third challenge was the limitation of the simulation temperature to a maximum of 500 K. This was done to prevent the potential desorption of the molecule from the surface. The original dataset was sampled at 500 K, which means it does not contain reference structures with large surface-molecule separations.

The fourth challenge extends the complexity of multi-component modeling by combining heavy and light atoms within one relatively large system. It is based on the Methylammonium (MA) Lead Iodide perovskite dataset. One can distinguish three types of interactions here: interatomic forces within MA molecules, interatomic forces between heavy I and Pb atoms, and the interaction between the MA and PbI_3_ subsystems. The additional complexity of the challenge arises from the sluggish Pb and I atoms' motion compared to that of H, C, and N atoms and the rotational freedom of the MA molecule within the PbI_3_ cage. All these lead to different requirements for the sufficient sampling of different system parts, further complicating the MLFF training process. The training and validation datasets are the same for all MLFFs; their sizes are 100, 200, 300, 400, and 500. Only the MLFF models trained on 500 reference structures were used for the stability and performance test similar to Challenge I with periodic boundary conditions in three dimensions.

It is worth noting that the datasets for all challenges can be categorized into two major groups: comprehensive datasets designed to cover all essential regions of the PES, including those for Challenges I (complete), II (complete), III, and IV; and partial datasets, which include the remaining datasets constructed to cover only specific regions of the PES, namely Challenges I (folded and unfolded) and II (incomplete and unknown).

## 4. Results and discussion

The results of the TEA Challenge 2023 are presented in two parts, the second of which can be found in ref. 66. This paper focuses on analysing MLFFs by evaluating their performance on test datasets and examining the basic outcomes of molecular dynamics (MD) simulations. Specifically, it presents the outcomes of the TEA 2023 in terms of Mean Absolute Errors (MAE), Root Mean Squared Errors (RMSE), and maximum errors (MAX) in energy and force predictions.

The MAX error for forces is computed as follows:1
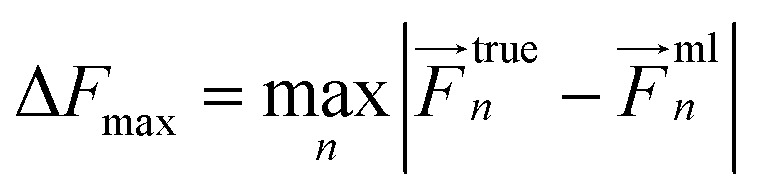
where *F*^true^_*n*_ is the reference force acting on an atom *n*, *F*^ml^_*n*_ is the corresponding force predicted by an ML model, |…| is the norm of a vector, and max is the maximum over atomic forces for all system configurations.

For the sake of completeness in the force analyses, we also provide values for forces acting on full molecules when they are system components. Namely, we include the total force acting on 1,8-Naphthyridine (Challenge III) and Methylammonium (Challenge IV) molecules2
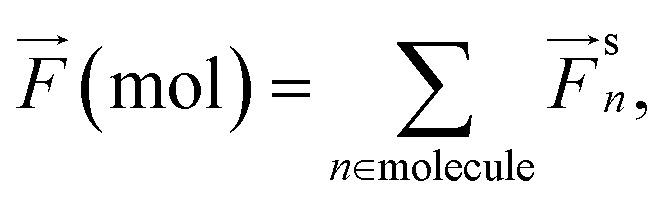
where *F*^s^_*n*_ denotes *F*^true^_*n*_ or *F*^ml^_*n*_. Since the PES is conservative, *F*(mol) deviates from zero only due to the interaction between the molecules and their environments.

To gain more detailed insight into MLFF performance, we go beyond the aggregate measures. A comprehensive analysis is carried out for atomistic force predictions by color coding the MAE for each individual atom in the system. Finally, to recognise practical considerations about the use of the MLFF models, the stability and computational speed of the MD simulations are also assessed. Detailed analyses of the MD results are published in Part II article of the TEA Challenge 2023.^66^

## 5. Aggregated accuracy

**Fig. 3:**
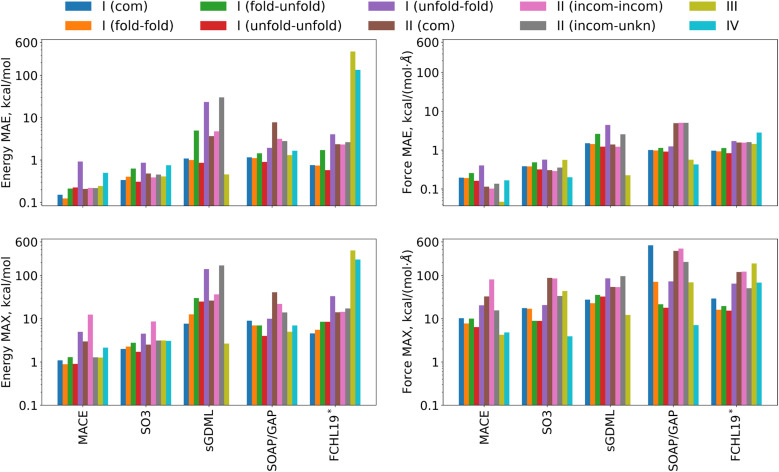
Graphical representation of MAE and MAX errors in energy and forces for the various challenges as listed in Table 1.

The first step in assessing the performance of the models in the TEA 2023 challenges is by considering the measures of aggregated accuracy as presented in Table 1. A comprehensive set of energy and forces MAE, RMSE, and MAX errors across all four challenges can be found there, with the MAE values below 1 kcal mol^−1^ or 1 kcal (mol^−1^ Å^−1^) highlighted in bold. To simplify the comparative analyses of the MLFF performance, the MAE and MAX energy and force errors are also plotted in Fig. 3, with the models' naming schemes detailed in the list of abbreviations. Tables SI 7 and SI 8 in the ESI† contain relative prediction errors normalized by the mean absolute values and the standard deviations of the energies and forces in the reference datasets.

**Table 1:** MAE, RMSE, and MAX errors, in that order, for energy and forces are reported in units of kcal mol^−1^ and kcal (mol^−1^ Å^−1^), respectively. Values in bold represent MAE below 1 kcal mol^−1^ and 1 kcal (mol^−1^ Å^−1^) for energies and forces, respectively

Challenge		MACE			SO3			sGDML			SOAP/GAP			FCHL19*		
I (com)	E	**0.15**	0.19	1	**0.34**	0.43	2	1.09	1.42	8	1.16	1.50	9	**0.76**	0.98	5
	F	**0.20**	0.30	10	**0.38**	0.57	18	1.50	2.14	28	1.01	1.67	500	**0.97**	1.36	29
	F(H)	**0.11**	0.17	9	**0.23**	0.33	12	**0.99**	1.32	22	**0.65**	0.87	23	**0.65**	0.88	29
	F(C)	**0.29**	0.40	9	**0.56**	0.76	18	2.13	2.81	26	1.41	2.21	500	1.36	1.80	22
	F(N)	**0.32**	0.44	10	**0.62**	0.83	10	2.57	3.39	28	1.46	2.77	490	1.42	1.85	17
	F(O)	**0.23**	0.32	9	**0.45**	0.60	14	1.39	1.87	22	1.31	1.70	19	1.07	1.41	20
I (fold–fold)	E	**0.12**	0.16	0.9	**0.40**	0.50	2	1.00	1.33	13	1.11	1.44	7	**0.74**	0.95	6
	F	**0.19**	0.29	8	**0.38**	0.55	17	1.43	2.05	23	**0.98**	1.39	71	**0.93**	1.30	16
	F(H)	**0.11**	0.17	7	**0.24**	0.34	17	**0.95**	1.27	16	**0.64**	0.86	20	**0.63**	0.85	16
	F(C)	**0.28**	0.39	6	**0.54**	0.73	8	2.04	2.72	23	1.37	1.84	71	1.31	1.72	15
	F(N)	**0.31**	0.43	7	**0.61**	0.81	11	2.39	3.18	23	1.38	1.83	71	1.37	1.78	16
	F(O)	**0.23**	0.32	8	**0.43**	0.58	11	1.29	1.76	20	1.26	1.65	25	1.01	1.33	11
I (fold–unfold)	E	**0.21**	0.28	1	**0.63**	0.75	3	4.96	7.01	30	1.44	1.80	7	1.72	2.24	9
	F	**0.26**	0.40	10	**0.48**	0.73	9	2.60	3.79	36	1.14	1.63	22	1.13	1.63	20
	F(H)	**0.14**	0.20	10	**0.28**	0.41	9	1.65	2.26	30	**0.71**	0.98	22	**0.73**	1.02	16
	F(C)	**0.39**	0.55	7	**0.70**	0.95	7	3.78	5.10	32	1.59	2.13	16	1.63	2.19	20
	F(N)	**0.42**	0.57	6	**0.82**	1.11	8	4.36	5.71	36	1.69	2.22	21	1.72	2.27	16
	F(O)	**0.34**	0.46	5	**0.61**	0.83	7	2.55	3.49	25	1.54	2.01	20	1.28	1.70	15
I (unfold–unfold)	E	**0.23**	0.26	0.9	**0.31**	0.39	2	**0.86**	1.43	25	**0.90**	1.17	4	**0.58**	0.81	9
	F	**0.16**	0.26	6	**0.32**	0.47	9	1.23	1.80	33	**0.92**	1.28	18	**0.83**	1.16	15
	F(H)	**0.10**	0.15	6	**0.20**	0.28	6	**0.83**	1.15	33	**0.61**	0.81	14	**0.58**	0.80	13
	F(C)	**0.23**	0.34	5	**0.45**	0.62	9	1.71	2.35	21	1.29	1.70	13	1.14	1.51	10
	F(N)	**0.26**	0.38	6	**0.51**	0.69	7	2.05	2.80	23	1.28	1.67	18	1.22	1.60	15
	F(O)	**0.19**	0.27	5	**0.36**	0.49	6	1.11	1.55	15	1.14	1.48	10	**0.90**	1.18	9
I (unfold–fold)	E	**0.92**	1.19	5	**0.86**	1.04	5	23.4	31.2	140	1.94	2.48	10	4.04	5.67	33
	F	**0.40**	0.74	21	**0.57**	0.87	21	4.43	6.66	86	1.25	1.85	73	1.71	2.62	65
	F(H)	**0.20**	0.35	20	**0.33**	0.52	21	2.95	4.62	86	**0.80**	1.22	73	1.18	1.93	65
	F(C)	**0.59**	0.99	14	**0.81**	1.13	18	6.00	8.22	75	1.65	2.25	48	2.11	2.93	36
	F(N)	**0.75**	1.07	21	**0.96**	1.28	12	7.34	9.74	58	1.96	2.63	50	3.03	4.17	30
	F(O)	**0.63**	0.99	12	**0.74**	1.02	19	4.96	7.12	53	1.75	2.40	25	2.04	2.91	49
II (com)	E	**0.21**	0.27	3	**0.48**	0.60	3	3.68	5.05	26	7.79	9.71	41	2.37	3.00	14
	F	**0.11**	0.17	33	**0.30**	0.46	88	1.39	2.06	54	4.93	6.80	370	1.57	2.18	120
	F(H)	**0.07**	0.10	33	**0.19**	0.28	57	**0.92**	1.30	54	3.41	4.63	230	1.05	1.41	120
	F(C)	**0.16**	0.22	10	**0.42**	0.59	16	1.90	2.62	36	7.04	9.10	110	2.22	2.90	81
	F(N)	**0.18**	0.25	24	**0.49**	0.67	88	2.40	3.28	47	6.25	8.03	230	2.14	2.75	59
	F(O)	**0.14**	0.20	22	**0.36**	0.50	43	1.34	1.89	37	4.91	6.43	370	1.69	2.22	35
II (incom–incom)	E	**0.22**	0.31	12	**0.39**	0.50	9	4.76	6.79	37	3.17	3.94	22	2.33	2.94	14
	F	**0.10**	0.16	81	**0.29**	0.44	85	1.22	1.85	54	5.04	6.93	420	1.56	2.17	120
	F(H)	**0.07**	0.12	81	**0.19**	0.29	85	**0.81**	1.17	54	3.47	4.70	340	1.04	1.41	120
	F(C)	**0.14**	0.20	17	**0.40**	0.56	36	1.67	2.35	31	7.29	9.35	100	2.20	2.87	84
	F(N)	**0.16**	0.24	34	**0.44**	0.63	52	2.10	2.93	32	6.35	8.23	420	2.13	2.75	59
	F(O)	**0.12**	0.18	35	**0.34**	0.49	79	1.18	1.71	49	4.75	6.14	93	1.69	2.23	110
II (incom–unkn)	E	**0.22**	0.28	1	**0.45**	0.58	3	30.2	35.4	170	2.81	3.47	14	2.64	3.37	17
	F	**0.14**	0.21	16	**0.36**	0.55	34	2.55	3.59	96	5.03	6.92	210	1.61	2.25	51
	F(H)	**0.08**	0.12	10	**0.21**	0.32	34	1.88	2.74	96	3.46	4.68	160	1.07	1.46	40
	F(C)	**0.19**	0.27	7	**0.51**	0.71	17	3.21	4.21	41	7.28	9.35	110	2.26	2.97	42
	F(N)	**0.23**	0.30	9	**0.58**	0.79	21	4.11	5.32	45	6.37	8.22	210	2.25	2.90	47
	F(O)	**0.18**	0.25	16	**0.45**	0.62	24	2.60	3.49	39	4.75	6.14	78	1.76	2.32	51
III	E	**0.24**	0.31	1	**0.41**	0.52	3	**0.46**	0.58	3	1.31	1.59	5	366	366	380
	F	**0.05**	0.08	4	**0.56**	0.93	44	**0.23**	0.37	12	**0.56**	0.76	69	1.44	1.91	190
	F(H)	**0.06**	0.09	2	**0.28**	0.40	9	**0.18**	0.26	3	**0.44**	0.63	25	1.29	1.70	134
	F(C)	**0.04**	0.07	4	**0.58**	0.95	44	**0.22**	0.37	12	**0.57**	0.75	69	1.43	1.89	190
	F(N)	**0.14**	0.20	3	**0.53**	0.75	13	**0.45**	0.70	12	**0.93**	1.23	21	2.21	2.92	90
	F(mol)	**0.12**	0.16	1	**0.58**	0.77	5	**0.16**	0.24	3	1.57	2.63	20	4.03	6.12	41
IV	E	**0.50**	0.65	2	**0.75**	0.94	3	—	—	—	1.66	2.09	7	134	138	240
	F	**0.17**	0.23	5	**0.20**	0.28	4	—	—	—	**0.43**	0.59	7	2.81	3.62	68
	F(H)	**0.12**	0.16	5	**0.13**	0.17	2	—	—	—	**0.29**	0.38	7	2.75	3.55	68
	F(C)	**0.18**	0.24	4	**0.19**	0.25	4	—	—	—	**0.50**	0.64	7	2.60	3.42	68
	F(N)	**0.21**	0.27	3	**0.23**	0.29	2	—	—	—	**0.54**	0.68	5	2.95	3.80	62
	F(Pb)	**0.28**	0.36	3	**0.41**	0.52	3	—	—	—	**0.83**	1.05	7	3.40	4.30	34
	F(i)	**0.20**	0.26	4	**0.26**	0.34	3	—	—	—	**0.50**	0.65	5	2.78	3.53	34
	F(mol)	**0.23**	0.29	4	**0.22**	0.28	2	—	—	—	**0.55**	0.70	5	6.23	7.92	53

The first conclusion that can be drawn from Table 1 is that the employment of equivariant NN architectures in MACE and SO3krates models significantly reduces MAE and RMSE for energy and forces throughout all tested systems compared to the kernel-based models: sGDML, SOAP/GAP, and FCHL19*. Traditionally, the benchmark for MLFF accuracy in quantum chemistry has been 1 kcal mol^−1^ MAE for energy and 1 kcal (mol^−1^ Å^−1^) MAE for forces. Equivariant architectures reduce typical MAEs by a factor of 2 to 5, depending on the number of parameters within the NN. This reduction represents a substantial leap in predictive accuracy, establishing new standards for force predictions in the range of 0.2–0.5 kcal (mol^−1^ Å^−1^).

Conversely, the standards for energy predictions have not seen a similar reduction. The increased size and complexity of the simulated systems complicate the reconstruction of global quantities, such as energy. This trend is evident when comparing the MAE and RMSE for the first two challenges, involving molecules with 42 and 100 atoms, against Challenge IV, where the unit cell consists of 384 atoms. While the MAE and RMSE for forces and energies are fairly similar for both molecules, the energy errors for MAPbI_3_ are 2.5–3.5 times larger compared to those for forces across all ML models capable of providing reliable predictions (MACE, SO3krates, and SOAP/GAP). Note however that since the total energy of a solid is an extensive quantity, even small errors per unit cell can be scaled to arbitrary values as the system size is scaled.

The second finding concerns the MAX errors. Despite the advancements in MLFF architectures, MAX errors remain substantial even for the most recent MLFF models. When trained on comprehensive (complete) datasets, the MACE and SO3krates models exhibit significant prediction discrepancies in the forces, with maximum MAX errors of 33 and 88 kcal (mol^−1^ Å^−1^), respectively. Moreover, the maximum MAX force error for the sGDML model is comparable at 54 kcal (mol^−1^ Å^−1^). Still, a significant improvement is observed compared to two other kernel-based models, SOAP/GAP and FCHL19*, which display considerably higher maximum MAX force errors of 500 and 190 kcal (mol^−1^ Å^−1^), respectively. On the other hand, SOAP/GAP generally displays smaller MAX energy errors than the other kernel models, indicating that the balance between energies and forces in the respective loss functions also plays a role here. The issue of large MAX errors can be further exacerbated when the MLFFs are trained on partial datasets (missing data). For the MACE model, the MAX error increases from 33 to 81 kcal (mol^−1^ Å^−1^), while for the sGDML model, it increases from 54 to 140 kcal (mol^−1^ Å^−1^).

The third observation is related to systems with prominent non-covalent binding. Particular attention should be paid to the total forces acting on the 1,8-Naphthyridine molecule in Challenge III and the Methylammonium molecule in Challenge IV. These forces arise from non-covalent interactions between the molecular and periodic components of the system, which are naturally weaker in magnitude compared to atom–atom interactions dominated by covalent bonding. Despite this, the MAE and RMSE for molecular forces are similar in magnitude to those for atomistic forces across all considered ML models. This is likely due to the fact that all loss functions contain total forces rather than non-covalent forces, and results in a higher relative error for non-covalent interactions, highlighting a critical area for enhancing ML architectures.

While for the organic systems of Challenges I and II equivariant NN architectures showed larger improvements, periodic systems could be more challenging for them as exemplified by the results of Challenge IV – arguably, the most complex one. For instance, the advantage of the MACE model, which in other cases exhibited MAE and RMSE values approximately half those of the SO3krates model and 5 to 10 times smaller values compared to kernel-based models, is only about 30% for the energy and just 15% for the forces compared with SO3krates. Also, the MAE and RMSE of the SOAP/GAP model are just twice as large as the NNs. While the difference in energy prediction accuracy was higher than for forces, it was also considerably reduced for all models except FCHL19* compared to Challenges I−III.

### 5.1. In-depth accuracy analysis

The analyses presented above follow the typical approach based on aggregated accuracy metrics. While that is a well-established method reliable for small and relatively simple systems in terms of chemical composition, it becomes insufficient as system size and composition diversity increase. In order to address this gap, the FFAST software^78^ is used to provide detailed performance quantifiers at the atomistic level.

**Fig. 4:**
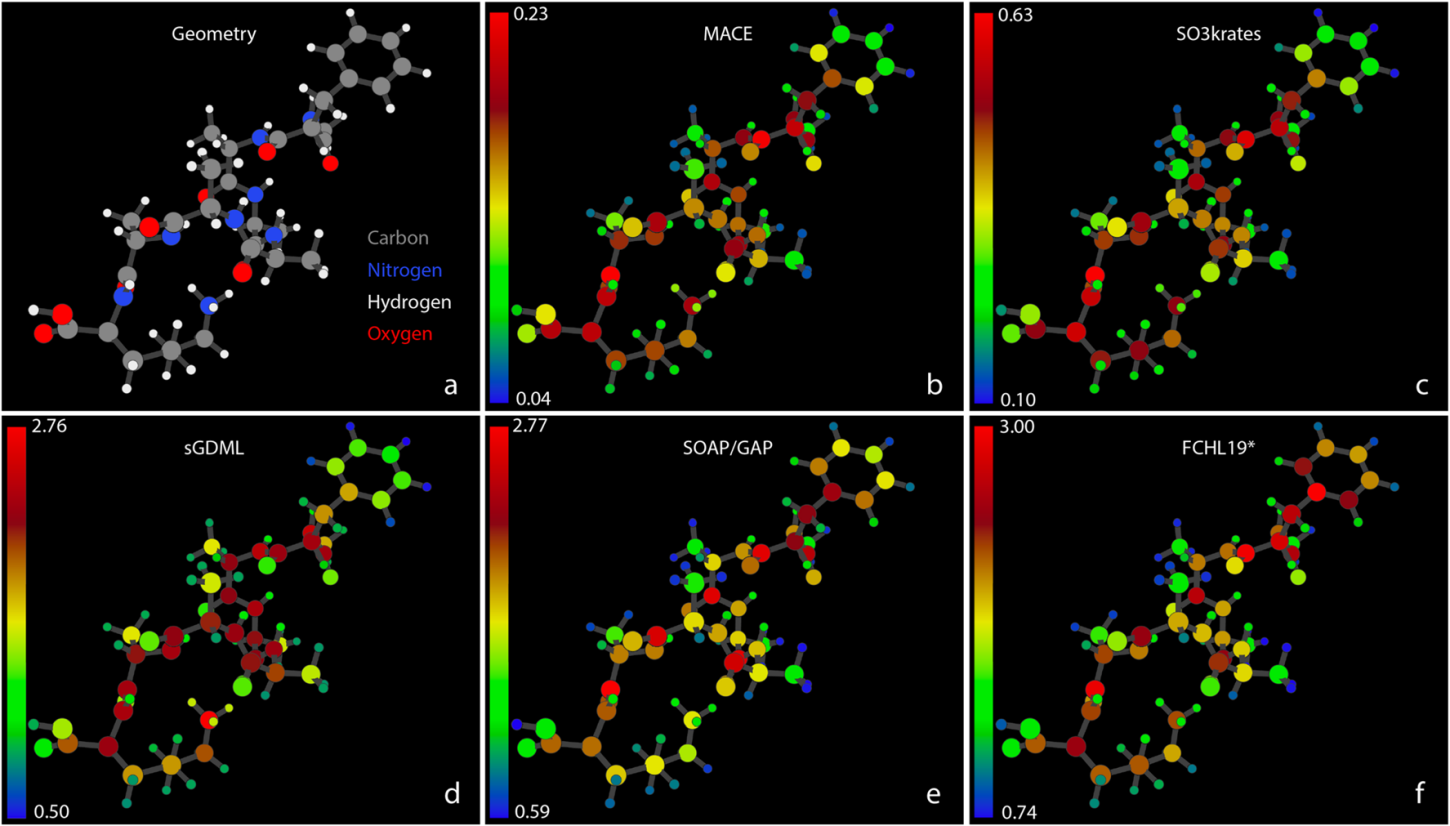
Atomic Force MAEs for Ac-Phe-Ala5-Lys. (a) A snapshot of the system geometry and atom types: carbons – grey, nitrogens – blue, hydrogens – white, and oxygens – red. (b)–(f) The MAEs for forces, measured in kcal (mol^−1^ Å^−1^), acting on individual atoms within the Ac-Phe-Ala5-Lys system. The MAEs correspond to the MLFF predictions on the test set and are represented with different colors according to the color bars shown with the corresponding scaling numbers, different for different MLFFs. Note the rather different absolute scale of the colour bars, ranging from 0.23 for MACE to 3.00 for FCHL19*.

Fig. 4 shows the MAEs of atomic forces for the Ac-Phe-Ala5-Lys molecule on the test set for all five MLFFs participating in the Challenge II. In Fig. 4b–f, atomic colors encode the force MAE for each individual atom, with corresponding color bars indicating numeric values. A similar Fig. SI 4† for the Ac-Ala3-NHMe molecule can be found in the ESI.† Note that individual colour scales are used for each model, and the absolute scale of the errors shown varies considerably between the models. In both figures, a significant heterogeneity in force prediction accuracy across the molecule can be seen. The MAE ratio between the worst and best-predicted atoms ranges from 6 for neural networks to 4–5 for kernel-based methods. Notably, even among atoms of the same type, such as carbons, the best-predicted atoms can have MAEs that are three times smaller than the worst-predicted carbons. This force prediction heterogeneity indicates that there is still room for improvement of MLFF architectures in achieving consistent accuracy across individual atoms.

Interestingly, the pattern of force prediction heterogeneity is very similar across all five MLFFs. The color bar values are non-withstanding; one could notice minimal differences between the relative errors of atom pairs, especially in the middle part of the molecule. Notable differences in prediction patterns appear mainly at the molecule's extremities. For example, examining the aromatic ring in the upper right corner of the figures, one can observe variations in accuracy for the C atoms, particularly those connecting the ring to the rest of the molecule, going from MACE or SO3krates MLFFs to sGDML, and finally to SOAP/GAP and FCHL19* models.

**Fig. 5:**
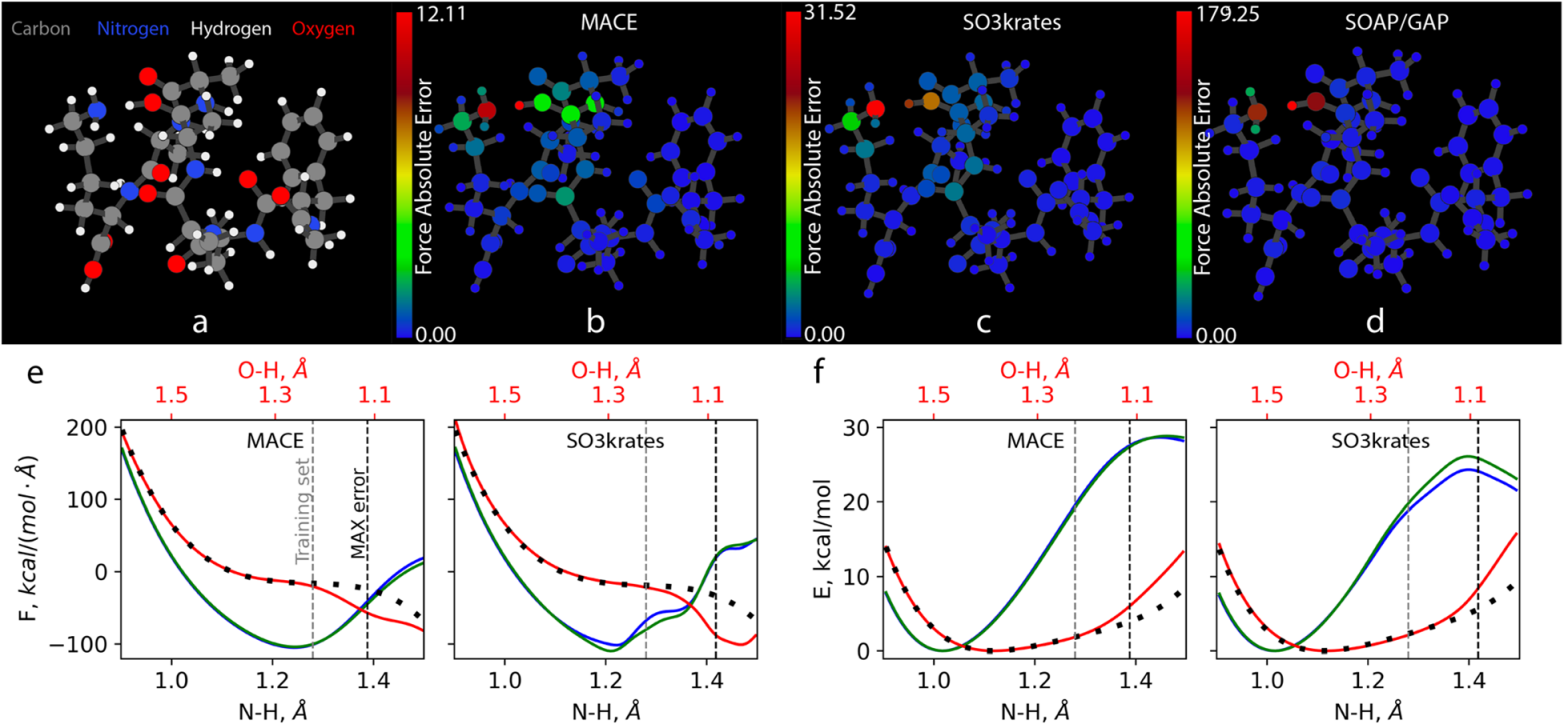
Maximum atomic force errors for Ac-Phe-Ala5-Lys. (a) A snapshot of the system geometry and atom types: carbons – grey, nitrogens – blue, hydrogens – white, and oxygens – red. (b)–(d) The absolute atomic force errors in kcal (mol^−1^ Å^−1^), as indicated by the corresponding colour. Note the different scales for the colour bars, ranging from 12.11 for MACE, 31.52 for SO3krates, to 179.25 for SOAP/GAP. (e) The projection of the force acting on hydrogen atoms along the vectors connecting the nitrogen and hydrogen atoms in the R-NH_3_ (with maximum prediction errors). These projections are shown as a function of the nitrogen–hydrogen distance. The solid lines represent the force predictions made by the MACE and SO3krates MLFFs, while the dotted lines correspond to the reference results obtained from DFT calculations. The red line depicts the hydrogen atom with the largest force prediction error. At the top of the figure, we also plot the distance between this hydrogen atom and the neighboring oxygen atom, which is only relevant for the red curve. (f) Illustration of the work required to move the hydrogen atom along bond vectors, with the energy minima used as reference zero points. Vertical dashed lines on (e) and (f) represent the longest NH bond distance found in the training set (grey) and the NH bond distance corresponding to the configuration with the maximum error (black). Blue and green solid curves show the PES scan for the two hydrogen atoms in the R-NH_3_ that move away from the molecule as the bond length increases (for comparison purposes).

The observed MAX error pattern in the MACE, SO3krates, and SOAP/GAP models again shows a similarity in the relative error between different atoms for these MLFFs. Fig. 5 illustrates the atomistic force errors for configurations from the test set where these models exhibit the most significant deviations from the reference data (Fig. SI 5–7† contain more examples from Challenges I–III). Although the three geometries are not identical, they represent consecutive points along one of the MD trajectories used to form the reference dataset. Note that our visualization software, which determines chemical bonds based on interatomic distances, erroneously depicts the hydrogen atom as being bonded to oxygen, whereas it is bonded to the nitrogen atom, as illustrated in Fig. 4 (see the NH_3_ tail in the lower central part).

**Fig. 6:**
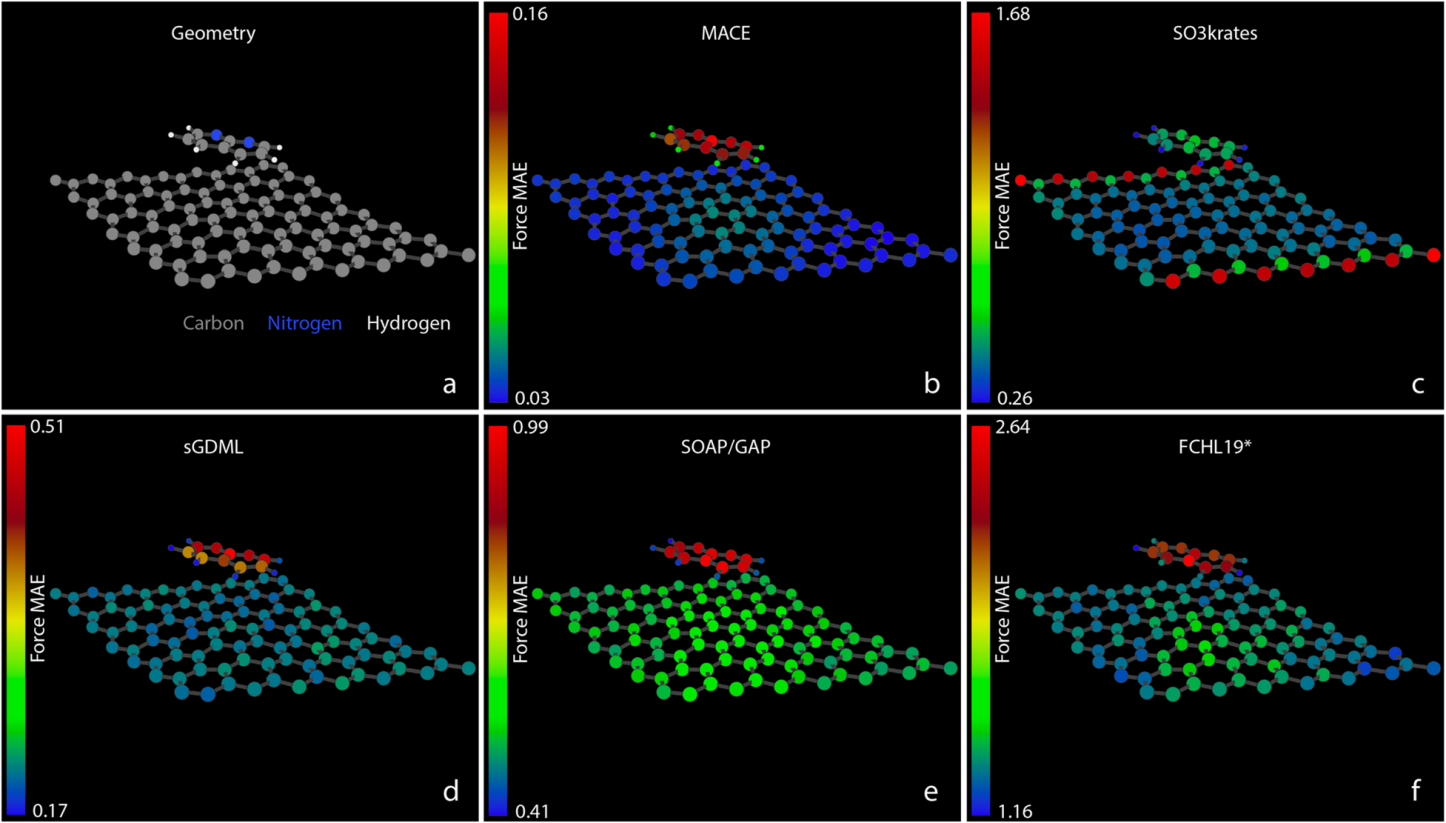
Atomic force MAEs for C_8_H_6_N_2_/C_98_. (a) A snapshot of the system geometry and atom types: carbons – grey, nitrogens – blue, and hydrogens – white. (b)–(f) The MAEs for forces, measured in kcal (mol^−1^ Å^−1^), acting on individual atoms within the C_8_H_6_N_2_/C_98_ system. The MAEs correspond to the MLFF predictions on the test set and are represented in different colors according to the colour bars shown with the corresponding scaling numbers, which are different for different MLFFs, with scales ranging from 0.16 for MACE to 2.64 for FCHL19*.

Fig. 6 presents a similar analysis of the MAEs for atomistic forces for the 1,8-Naphthyridine molecule adsorbed on a graphene test set of Challenge III. While there is a vast difference between the absolute accuracy of the best (MACE) and the worst (FCHL19*) models, all MLFFs, except SO3krates, exhibit similar patterns of relative force accuracy between different atoms. The H atoms and C atoms in graphene are treated with relative ease, while C and N atoms in the 1,8-Naphthyridine molecule show significantly larger force errors. The variation of atomic force error is particularly pronounced for the MACE model, where the MAEs for C atoms in graphene are five times smaller than those for the C atoms in the 1,8-Naphthyridine molecule. Interestingly, the exceptionally high accuracy of force reconstruction within the graphene layer, with an MAE of approximately 0.03 kcal (mol^−1^ Å^−1^), results in a noticeable ‘shadow’ of the molecule on the graphene surface in Fig. 6b. This ‘shadow’ represents the region most affected by molecule–surface interactions where the MACE model exhibits lower accuracy compared to regions away from the molecule. This observation aligns with the aforementioned challenging nature of non-covalent interactions. This effect is not seen for other models whose errors on the carbon atoms in the graphene sheet are much higher thus masking the above effect.

The significant errors for C atoms on the two borders of the graphene cell produced by the SO3krates model are unexpected and due to precision design choices employed in the JAX ecosystems for certain GPU hardware (see explanation below). It further does not affect the faithfulness of simulation and derived observables. It is important to note that only the visualisation of atomistic errors allows for identifying subtle but essential prediction problems. This underscores the necessity of employing software packages such as FFAST, which are designed for comprehensive MLFF performance analysis, to ensure the reliability of the predicted forces and the results of consequent simulations. The cause of the errors for SO3krates is due to the JAX default of running in tensorflow32 precision on A100 and H100 GPU. This problem can be alleviated by explicitly disabling tensorflow32 precision (which performs some operations in float16) and instead running training and evaluation in standard float32 precision. This not only alleviates the boundary effects (ESI Fig. SI 8†) but also gives significantly lower errors, ranging from 0.09 to 0.32 kcal (mol^−1^ Å^−1^).

**Fig. 7:**
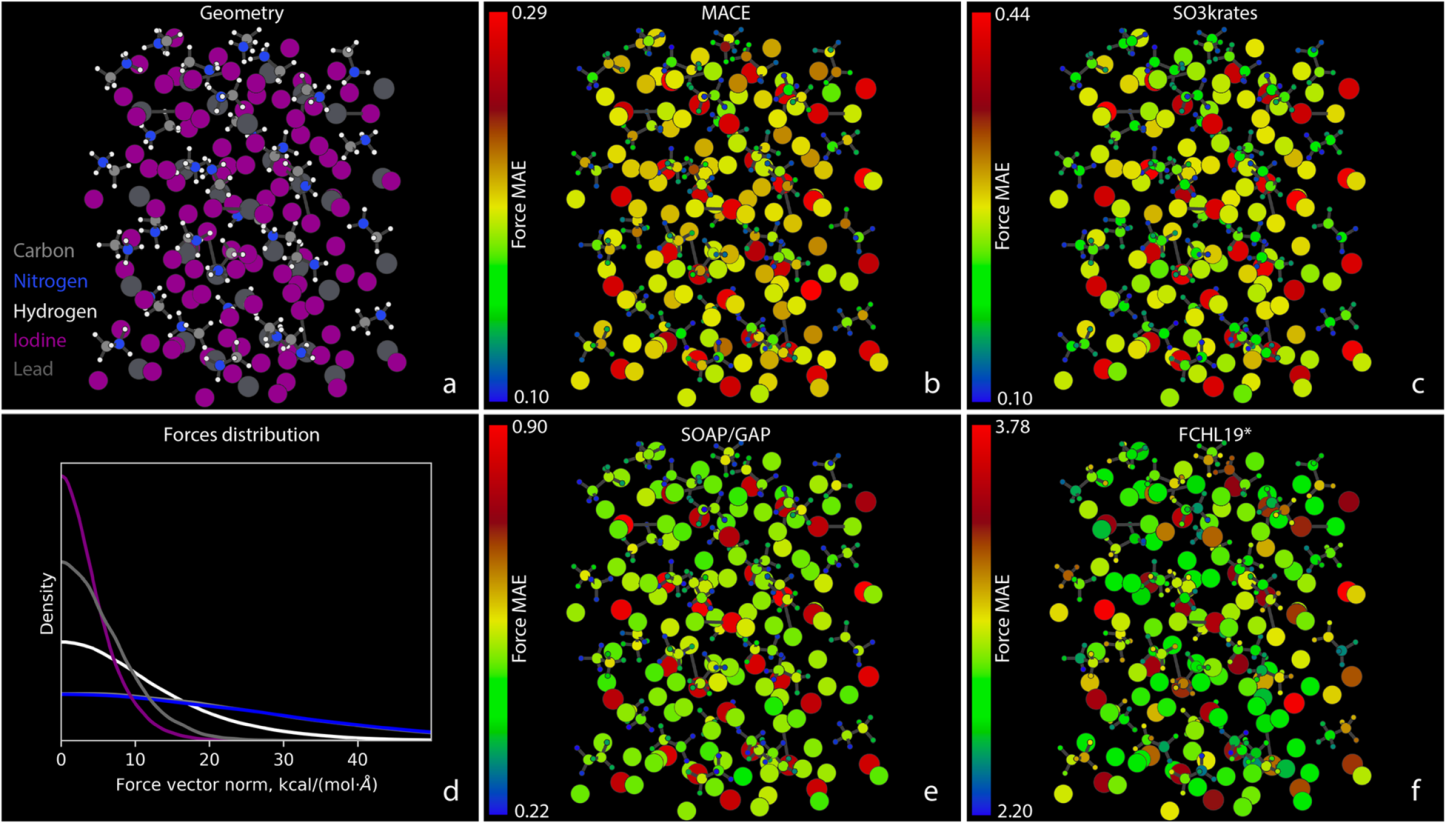
Atomic force MAEs for MAPbI_3_. (a) A snapshot of the system geometry and atom types: carbons – grey, nitrogens – blue, hydrogens – white, iodines – purple, and leads – dim grey. (b), (c), (e), and (f) The MAEs for forces, measured in kcal (mol^−1^ Å^−1^), acting on individual atoms within the MAPbI_3_ system. The MAEs correspond to the MLFF predictions on the test set and are represented in different colors according to the color bars shown with the corresponding scaling numbers, which are different for different MLFFs. (d) Illustration of the distribution of the norms of atomic force vectors for different chemical elements. The carbon curve lies under the nitrogen one, as expected due to the Newton's 3^rd^ law.

The observations made thus far are also consistent with the results of Challenge IV, as presented in Fig. 7 for the atomic force MAEs for MAPbI_3_. Similar force prediction heterogeneity patterns are observed across all MLFFs. Fig. 7b, c and e appear nearly identical when disregarding the color bar values that indicate the vast difference between absolute errors. Surprisingly, the most problematic atoms for all MLFFs are the Pb atoms. The largest MAEs for these atoms anti-correlate with the strength of the forces they experience. This can be seen in Fig. 7d, which illustrates the distribution of the magnitudes of atomic forces for different chemical elements in the system. The forces on Pb atoms, shown in dim grey, are the second smallest, only slightly larger than those on I atoms. Meanwhile, the MAEs for force reconstruction on I, C, N, and H atoms throughout the system are more than twice as small as those for Pb atoms. This clearly indicates the need for improvements in either MLFF architectures, loss functions, or training datasets. As we will demonstrate in Part II of this manuscript, the large MAEs for Pb atoms directly impact the stability of MD simulations.^66^

Finally, in Challenge IV, we observe only a minor difference in accuracy for the forces between MACE and SO3krates, although the energy error of MACE is 30% lower. This suggests that, beyond a certain level of system complexity, integrating even more principles of chemistry and physics is necessary for more accurate and reliable modeling. Therefore, despite the tremendous progress in MLFF architectures over the last decade, there remains room for improvement and further developments.

In summary, novel equivariant MLFF architectures like MACE and SO3krates significantly enhance the faithful reconstruction of potential energy surfaces for molecules, molecular interfaces, and periodic systems. However, a major challenge remains in eliminating rare but substantial errors in force predictions. The observed maximum force errors are comparable to those of previous MLFF generations, such as sGDML. Another area for improvement is the description of non-covalent interactions, which are often obscured by strong covalent bonding. Additionally, force reconstruction demonstrates significant heterogeneity between different atoms of a given system that is consistent across all tested models, including both kernel-based and neural network approaches. The consistent error patterns are primarily due to the L2 nature of the loss function applied to the forces vector across all models. However, the observed heterogeneity, which does not always align with the magnitude of the force acting on an atom, suggests a direction for further development of MLFFs. We also emphasize the need to move beyond aggregate accuracy measurements, such as system MAE or RMSE, to uncover potential pitfalls of MLFFs.

### 5.2. Stability and speed

One of the critical requirements for any MLFF is the ability to run efficient and reliable simulations. Beyond achieving low MAE, RMSE, or MAX errors on test sets, an MLFF must also ensure stable and computationally efficient MD trajectories. To assess this, we conducted 12 independent MD simulations, each for one million steps, using all MLFF architectures participating in the TEA Challenge 2023. While a detailed analysis of the resulting trajectories is presented in a companion paper,^66^ we focus here on stability analyses and the averaged computational times required to generate 1 ns of dynamics (one million energy and force evaluations).

Table 2 provides a summary of the stability test results. Stability was assessed using a broken bond criterion, terminating the simulation if any covalent chemical bond in the system exceeded a length of 2 Å. Under the given simulation conditions, the test systems were not expected to undergo proton transport or other processes that would alter their bonding patterns.

**Table 2:** Molecular dynamics stability in the *A*/*B* ratio. Here, *A* is the number of completed trajectories (1 ns), and *B* is the number of failed trajectories (broken chemical bonds)

Simulation		Challenge						
Model	Temperature, K	I			II		III	IV
		Com	Fold	Unfold	Com	Incom		
MACE	300	12/0	12/0	12/0	12/0	12/0	12/0	12/0
	500	12/0	12/0	12/0	12/0	12/0	12/0	0/12
	700	12/0	12/0	12/0	12/0	12/0	—	—
SO3krates	300	12/0	12/0	11/1	12/0	12/0	12/0	12/0
	500	12/0	11/1	5/7	12/0	12/0	6/6	10/2
	700	4/8	7/5	0/12	5/7	8/4	—	—
sGDML	300	12/0	12/0	0/12	12/0	12/0	12/0	—
	500	7/5	7/5	0/12	12/0	12/0	12/0	—
	700	—	—	—	12/0	12/0	—	—
SOAP/GAP	300	2/10	0/12	8/4	0/12	0/12	2/10	0/12
FCHL19*	300	0/12	0/12	0/12	0/12	0/12	1/11	0/12

The MACE architecture demonstrated the highest stability in our tests, successfully completing most MD trajectories. It only failed in the MAPbI_3_ system at 500 K, where it could not provide a single 1 ns long MD trajectory. The second most stable architecture, SO3krates, performed well in this scenario, successfully completing 10 out of 12 MD runs. However, SO3krates faced stability issues in most of the other challenges when the temperature increased to 500 K and above. The causes of the difference in stability between the equivariant NN architectures is unclear at the moment and requires further investigation.

Among the kernel-based models, the sGDML model exhibited the highest stability. It performed well at 300 K across all challenges except Challenge I, which had a significantly incomplete training set. It also maintained stability in Challenges II and III at 500 K. Nevertheless, the limited sampling of the PES in the reference dataset for Challenge I led to instability in the MD simulations of the Alanine tetrapeptide molecule at 500 K.

The SOAP/GAP and FCHL19* models were quite unstable. These models struggled to provide stable dynamics across all challenges, with some simulations failing within just a few thousand steps. This is unsurprising as these kinds of kernel models typically need to be trained in active learning workflows to achieve stable dynamics.^9,79–81^ Nevertheless, achieving simulation durations ranging from dozens to even hundreds of picoseconds is feasible using the SOAP/GAP and FCHL19* models, particularly in well-sampled, near-equilibrium PES regions.

Overall, our results highlight the varying degrees of stability among different MLFF architectures and emphasize the need for continued improvements to ensure reliable MD simulations, *cf.* also.^62,82^ Additionally, the MACE and SO3krates models, when trained on the unfolded Alanine tetrapeptide and MAPbI_3_ datasets, highlight an important observation. Despite the fact that the mean and maximum force errors of these models were much lower than those of the kernel models, this did not necessarily translate to better stability of MD simulations at 500 K, for the case of SO3krates. This suggests a weak correlation between the accuracy of MLFFs on the test set and their reliability in actual simulations. This finding indicates that high aggregate accuracy on reference data does not necessarily translate to stable and reliable performance in practical MD simulations. On the other hand, the models with the most accurate aggregate statistics (MACE and SO3krates) also consistently exhibit the highest stability. Therefore, overall accuracy is necessary for stability but not sufficient on its own.

Building on this, incorporating uncertainty estimations into the workflow will represent a crucial step in enhancing MLFF modeling reliability. For kernel-based models, Bayesian methods provide a relatively straightforward way to achieve this. However, NN models such as MACE and SO3krates currently lack intrinsic mechanisms for estimating prediction uncertainty. Meanwhile, uncertainty estimation in NNs has been a widely researched topic in other fields.^83^ Adapting and integrating the developed methodologies into MLFF codes should become a key research focus, especially as the complexity and scale of target systems grow, making reference calculations computationally prohibitive.

Table 3 summarizes the average computational time required for each ML model to produce 1 ns of MD dynamics (1 million steps) on a single NVIDIA A100-40 GPU. The reported times are averaged across all MD trajectories within each challenge. All run conditions and scripts were identical for all models except for the ASE calculator and the specific ML architecture provided by the participant.^84^ It is important to note that the SOAP/GAP models cannot be executed on a GPU. Instead, these models were run on CPU nodes consisting of 2 AMD Rome CPUs, each with 64 cores at 2.6 GHz, for a total of 128 cores and 512 GB of RAM. This hardware configuration was used to ensure that SOAP/GAP models could complete the MD simulations within a reasonable timeframe.

**Table 3:** Average simulation time in hours per 1 ns of molecular dynamics. The bold font indicates models that failed to generate a single 1 ns MD trajectory

Simulation	Challenge			
Model	I	II	III	IV
MACE	34.4	43.6	45.4	36.1
SO3krates	1.4	2.0	4.2	6.7
sGDML	2.4	2.7	224.8	—
SOAP/GAP	83.6	**111.0**	64.9	**606.2**
FCHL19*	**11.9**	**12.4**	9.9	**29.2**

We found that the SO3krates model, with its current settings, was the fastest in production, outperforming its NN competitor, MACE, by a factor of 6 to 25, depending on the system size. Surprisingly, it was even faster than the sGDML model, known for its minimalistic architecture, which ranked second in running MD simulations for molecules from Challenges I and II. However, for larger systems, such as the molecule–surface interface in Challenge III, the need to explicitly account for permutational symmetries significantly decreased the efficiency of the sGDML model, making it the slowest among all TEA 2023 competitors. The third fastest ML model in production was FCHL19*, but this came at the cost of the largest instability in MD simulations. The MACE architecture, with its current settings, ranked fourth. For Challenges I, II, and III, MACE showed only a 1.5 to 2.5 times increase in prediction speed compared to the SOAP/GAP model, even though SOAP/GAP ran on CPU rather than GPU. However, for the largest test system, MAPbI_3_, the speed difference between MACE and other competitors, namely SO3krates and FCHL19*, became smaller. Although the system sizes in the TEA 2023 Challenge were relatively small compared with those needed and used in most scientific studies using molecular dynamics, it is apparent that in this regime the relationship between the simulation time and system size can be complicated, suggesting a need for further code optimization. Increasing the system size may result in a shift from a memory-bound to a compute-bound regime, further complicating performance analysis.

### 5.3. Pitfalls

When utilizing any modern MLFF, it is important to recognize that much of the software is developed by scientists with varying degrees of coding experience, and not professional software engineers. Below, we outline some of the issues the TEA team encountered in the process of installation, integration within common frameworks, and during MD runs and tests with various MLFF architectures:

(1) Compatibility issues: models trained with previous MLFF versions may not be supported after software updates, potentially leading to incorrect results.

(2) Lack of update information: information regarding updates is often missing, making it difficult to track changes and their impacts.

(3) Inconsistent standards: there are no universally accepted standards for units, inputs, and outputs, leading to inconsistencies across different implementations.

(4) Installation and benchmarking challenges: software installation and performance benchmark tests are typically absent, meaning there is no assurance that the MLFF will function as expected in your specific environment.

(5) Systematic offsets communication: the presence of systematic offset (*e.g.* for the energy prediction) can be a challenge to obtaining correct results when these systematic offsets are not clearly communicated in the software itself or its manual.

Several such pitfalls were corrected in the course of the challenge, highlighting the benefits such collaborative efforts bring to the entire community.

## 6. Conclusions

The TEA Challenge 2023 provided a comprehensive evaluation of a representative sample of modern MLFF models, capturing the current state of the field and identifying the strengths and weaknesses of existing architectures. ML models were tested for their ability to predict potential energy surfaces and forces with high accuracy and reliability. The selected models included both kernel and NN architectures, namely MACE, SO3krates, sGDML, SOAP/GAP, and FCHL19*, representing the MLFF developers who participated in TEA 2023. The evaluation comprised four distinct tasks, each designed to test the limits of MLFFs under various conditions and system complexities. The performance of MLFF models is inherently dependent on the quality of the reference data. Therefore, we emphasize that all conclusions drawn in this manuscript are specific to the reference data computed at the PBE(0) MBD(–NL) level of theory. This level of theory is among the most accurate options currently available for generating sufficient reference data for the system sizes and complexities addressed in this study. Another test case for MLFFs, namely a compressed hydrogen benchmark dataset is available in the ESI.† Limited tests were also performed for the PhysNet model as detailed in the ESI.†

The results indicated significant advancements in the accuracy of MLFFs, particularly with novel equivariant neural network architectures such as MACE and SO3krates. These models showed marked improvements in MAE and RMSE for energy and force predictions compared to kernel-based models like sGDML, SOAP/GAP, and FCHL19* in the framework of the TEA 2023 challenges with force prediction accuracy in the range of 0.2–0.5 kcal (mol^−1^ Å^−1^).

However, maximum errors remain a critical challenge, highlighting the necessity for further refinement to reduce rare but substantial prediction discrepancies. Additionally, all ML models demonstrated significant heterogeneity in force error across the different atoms in a given system, with a worst-to-best atomistic MAE ratio of 5 : 1–6 : 1. Interestingly, the heterogeneity patterns were consistent across all MLFF architectures.

We also observed a trend of reduced prediction accuracy for non-covalent forces in favor of minimizing errors for strong covalent interatomic bonding. Atomistic MAE did not always correlate with the strength of the forces experienced by the atoms.

For Challenge IV, the MAPbI_3_ application, only a minor force accuracy difference of 0.03 kcal (mol^−1^ Å^−1^), was observed between the MACE and SO3krates models, whereas the energy accuracy of MACE was 30% higher. We note that to fully understand these differences, accuracy as a function of model size needs to be characterised. Making this tradeoff is a significant choice facing modelers, and exploring this aspect of MLFF construction is an important avenue of research for the future. Advancing the field could also be done by incorporating more physical principles into ML models, particularly for the description of long-range non-covalent interactions and strong short-range repulsive forces, which are often poorly represented in typical training sets or obscured by dominant covalent bonding effects.

Stability assessments revealed that the MACE and SO3krates models generally exhibited better stability in molecular dynamics simulations, completing most trajectories under varying conditions. Conversely, kernel-based models like SOAP/GAP and FCHL19* showed substantial instability, particularly at higher temperatures, though they performed adequately in well-sampled, near-equilibrium potential energy surface regions.

All the model architectures offer opportunities for trade-offs between model accuracy and computational efficiency, and we already noted in the Introduction that participants were left to make their own choices regarding this trade-off. Given these individual choices, in terms of computational speed, the SO3krates model fitted here outperformed its competitors by a significant margin (6 times or more), demonstrating the potential for highly efficient molecular dynamics simulations. Overall, MLFF performances may vary by two orders of magnitude for the same task and even within the same class of ML models, *e.g.* MACE and SO3krates, prediction speed can differ by a factor of 25. In addition to potential code optimisation, for each architecture, speed is related to model size, and to properly compare different architectures, the above-mentioned tradeoff between model size, corresponding speed, and prediction accuracy needs to be explored.

We also identified several technical pitfalls encountered during the TEA Challenge 2023. While direct communication with MLFF architecture developers allowed us to resolve most issues, this may not be available for ordinary users to such an extent.

Overall, the TEA Challenge 2023 demonstrated notable progress in MLFFs over the past decade, achieving new standards for accuracy and stability. However, continued innovation and optimization are essential to fully realize the potential of MLFFs in practical applications, ensuring reliable and efficient simulations across a broad spectrum of chemical systems. An in-depth analysis and discussion of the MD simulation results is available in the follow-up paper “Crash testing machine learning force fields for molecules, materials, and interfaces: molecular dynamics in the TEA Challenge 2023”.^66^

## Abbreviations

(1) SO3: SO3krates
(2) sGDML: Symmetric Gradient Domain Machine Learning
(3) SOAP/GAP: Smooth Overlap of Atomic Position/Gaussian Approximation Potential
(4) FCHL19*: Faber–Christensen–Huang–Lilienfeld 2019
(5) FFAST: Force Field Analysis Software and Tools
(6) MAE: Mean Absolute Error
(7) MAX: Maximum Absolute Error
(8) RMSE: Root Mean Square Error
(9) (in)com: (in)complete
(10) (un)fold: (un)folded
(11) unkn: unknown

## Data availability

All the TEA 2023 datasets are in the Zenodo archive https://doi.org/10.5281/zenodo.14138387.

## Author contributions

Igor Poltavsky – conceptualization, data curation, formal analysis, funding acquisition, methodology, project administration, software, supervision, validation, visualization, writing – original draft, writing – review & editing. Anton Charkin-Gorbulin – data curation, formal analysis, investigation, software, validation, visualization, writing – original draft, writing – review & editing. Mirela Puleva – data curation, formal analysis, investigation, project administration, software, validation, visualization, writing – original draft, writing – review & editing. Grégory Fonseca – data curation, formal analysis, investigation, software, validation, visualization, writing – original draft, writing – review & editing. Ilyes Batatia – data curation, formal analysis, methodology, software, writing – review & editing. Nicholas J. Browning – data curation, formal analysis, writing – review & editing. Stefan Chmiela – funding acquisition, supervision, writing – review & editing. Mengnan Cui – data curation, formal analysis, methodology, funding acquisition, writing – review & editing. J. Thorben Frank – data curation, formal analysis, funding acquisition, methodology, software, writing – review & editing. Stefan Heinen – data curation, formal analysis, methodology, software, writing – review & editing. Bing Huang – data curation, formal analysis, methodology, software, writing – review & editing. Silvan Käser – data curation, formal analysis, methodology, software, writing – review & editing. Adil Kabylda – data curation, formal analysis, methodology, software, writing – review & editing. Danish Khan – data curation, formal analysis, methodology, writing – review & editing. Carolin Müller – data curation, funding acquisition, supervision, writing – review & editing. Alastair J. A. Price – data curation, formal analysis, methodology, software, writing – review & editing. Kai Riedmiller – data curation, formal analysis, funding acquisition, writing – review & editing. Kai Töpfer – data curation, formal analysis, methodology, software, writing – review & editing. Tsz Wai Ko – writing – review & editing. Markus Meuwly – funding acquisition, resources, supervision, writing – review & editing. Matthias Rupp – data curation, writing – review & editing. Gabor Csányi – funding acquisition, resources, supervision, writing – review & editing. O. Anatole von Lilienfeld – funding acquisition, resources, supervision, writing – review & editing. Johannes T. Margraf – funding acquisition, resources, supervision, writing – review & editing. Klaus-Robert Müller – funding acquisition, resources, supervision, writing – review & editing. Alexandre Tkatchenko – conceptualization, funding acquisition, methodology, project administration, resources, supervision, writing – review & editing.

## Conflicts of interest

GC has equity interest in Symmetric Group LLP that licenses force fields commercially and also in Ångström AI, Inc.

## Supplementary Material

SC-016-D4SC06529H-s001
