# Supplementary material for: Crash testing machine learning force fields for molecules, materials, and interfaces: model analysis in the TEA Challenge 2023
Source: Chem Sci. 2025 Feb 10;16(8):3720–37. doi: 10.1039/d4sc06529h (PMC11809572; doi:10.1039/d4sc06529h)
Supplement: SC-016-D4SC06529H-s001 [file SC-016-D4SC06529H-s001.pdf]

Supplementary Information (SI) for Chemical Science.
This journal is © The Royal Society of Chemistry 2025
**These authors contributed equally to the work.

SI: Crash Testing Machine Learning Force Fields for Molecules, Materials, and Interfaces: Model Analysis in the TEA Challenge 2023

Igor Poltavsky*[1], Anton Charkin-Gorbulin**[2], Mirela Puleva**[3], Grégory Cordeiro Fonseca**[4], Ilyes Batatia[5], Stefan Chmiela[6], Mengnan Cui[7], J. Thorben Frank[8], Stefan Heinen[9], Nicholas J. Browning[10], Bing Huang[11], Silvan Käser[12], Adil Kabylda[13], Danish Khan[14], Carolin Müller[15], Alastair J. A. Price[16], Kai Riedmiller[17], Kai Töpfer[18], Tsz Wai Ko[19], Markus Meuwly[20], Matthias Rupp[21], Gabor Csanyi[22], O. Anatole von Lilienfeld[23], Johannes T. Margraf[24], Klaus-Robert Müller[25], and Alexandre Tkatchenko*[26]

[1,2,3,4,13,26]Department of Physics and Materials Science, University of Luxembourg, L-1511 Luxembourg, Luxembourg.
[2]Laboratory for Chemistry of Novel Materials, University of Mons,B-7000 Mons, Belgium.
[3,26]Institute for Advanced Studies, University of Luxembourg, L-1511 Luxembourg, Luxembourg.
[5,22]Department of Engineering, University of Cambridge, Trumpington Street, Cambridge, CB2 1PZ, United Kingdom.
[6,8,25]Machine Learning Group, Technical University Berlin, Berlin, Germany.
[6,8,25]BIFOLD, Berlin Institute for the Foundations of Learning and Data, Berlin, Germany.
[7]Fritz-Haber-Institut der Max-Planck-Gesellschaft, Berlin, Germany.
[9,14,23]Vector Institute for Artificial Intelligence, Toronto, ON, M5S 1M1, Canada.
[10]Swiss National Supercomputing Centre (CSCS), 6900 Lugano, Switzerland.
[11]Wuhan University, Department of Chemistry and Molecular Sciences, 430072 Wuhan, China.
[12,20,18]Department of Chemistry, University of Basel, Klingelbergstrasse 80, CH-4056 Basel, Switzerland.
[14]Chemical Physics Theory Group, Department of Chemistry, University of Toronto, St. George Campus, Toronto, ON, Canada.
[15]Friedrich-Alexander-Universität Erlangen-Nürnberg, Computer-Chemistry-Center, Nägelsbachstraße 25, 91052 Erlangen, Germany.
[16,23]Department of Chemistry, University of Toronto, St. George campus, Toronto, ON, Canada.
[16,23]Acceleration Consortium, University of Toronto. 80 St George St, Toronto, ON M5S 3H6, Canada.
[17]Heidelberg Institute for Theoretical Studies, Heidelberg, Germany.
[19]Department of NanoEngineering, University of California San Diego, 9500 Gilman Dr, Mail Code 0448, La Jolla, CA 92093-0448, United States.
[21]Luxembourg Institute of Science and Technology (LIST), L-4362 Esch-sur-Alzette, Luxembourg.
[23]Department of Materials Science and Engineering, University of Toronto, St. George campus, Toronto, ON, Canada.
[23]Department of Physics, University of Toronto, St. George campus, Toronto, ON, Canada.
[23]Machine Learning Group, Technische Universität Berlin and Berlin Institute for the Foundations of Learning and Data, Berlin, Germany.
[24]University of Bayreuth, Bavarian Center for Battery Technology (BayBatt), Bayreuth, Germany
[25]Department of Artificial Intelligence, Korea University, Seoul, South Korea
[25]Max Planck Institut für Informatik, Saarbrücken, Germany
[25]Google DeepMind, Berlin, Germany
[1,26]Corresponding authors: alexandre.tkatchenko@uni.lu, igor.poltavskyi@uni.lu

November 28, 2024

1 Methods

Details on the methods used in the TEA Challenge 2023 can be found here.

1.1 MACE

All MACE models trained in the paper use the MACE [1] architecture implemented in PyTorch and employing the e3nn library. The MACE training and evaluation are available at https://github.com/ACEsuit/mace/. MACE is an equivariant message-passing graph neural network where each layer encodes many-body information of atomic geometry. At each layer, manybody messages are formed using a linear combination of a tensor product basis [2]. This is constructed by taking tensor products of a sum of two-body permutation-invariant polynomials, expanded in a spherical basis. The final output is the energy contribution of each atom to the total potential energy. For a more detailed description of the architecture, see Refs. [1] and [3].

All MACE models use two MACE layers, with a spherical expansion of up to $l_{\max} = 3$, and 4-body messages in each layer (correlation order 3). We use a self-connection for both layers, a 128-channel dimension for tensor decomposition and a radial cutoff of 6 Å. We expand the interatomic distances into 8 Bessel functions multiplied by a smooth polynomial cutoff function to construct radial features, which in turn fed into a fully connected feed-forward neural network with three hidden layers of 64 hidden units and SiLU non-linearities. A maximal message equivariance of $L = 2$ is applied. The irreducible representations of the messages have alternating parity (in *e3nn* notation, `128x0e + 128x1o + 128x2e`).

The models are trained with the AMSGrad variant of Adam with default parameters. We use a learning rate of 0.01 and an exponential moving average (EMA) learning scheduler with decaying factor of 0.995. We use a batch size of 5, and we decrease the learning scheduler on a plateau with a factor of 0.8. The models are trained until no improvement is observed for 200 epochs. The models were trained on Nvidia A100 80GB GPUs. Total training time varies from 30 minutes for the smallest sets of Challenge I, to 32 hours for the largest sets of tasks III and IV.

1.2 SO3krates

We used the SO3krates network [4, 5] for training on the different challenges from the TEA23 workshop. The SO3krates network is an equivariant message-passing neural network, which replaces SO3 convolutions with an equivariant attention mechanism that is defined on the invariant output of SO3 convolutions over equivariant atomic representations. This allows to combine data efficiency, accuracy and stability with small computational cost.

All SO3krates models are trained on a combined loss of energy and forces with a weighting factor of 1000 to 1 between forces and energy. We used the ADAM optimiser with an initial learning rate of 1e-3 and an exponential learning rate decay every 100k steps by a factor of 0.7. Each model is trained for 1M gradient training steps and with a batch size of 1 for Challenge I, with a batch size of 10 for Challenge II and with a batch size of 10 for Challenge III and batch size of 2 for challenge IV. All used SO3krates models use a feature dimension of 128 and euclidean variables {1, 2, 3, 4} as well as three message passing layers. For Challenges II and III we used the same model as for Challenge I, with an additional residual MLP after the attention block and the update block in each layer. The average time per training gradient step for challenge I is 10 ms, for challenge II it is 10 ms, for challenge III it is 15 ms and 27 ms for challenge IV. All training runs were performed on an NVIDIA A100 40GB GPU.

1.3 sGDML

Symmetric Gradient Domain Machine Learning (sGDML) is a kernel-based method designed to efficiently reconstruct accurate molecular force fields from reference datasets obtained through high-level ab initio calculations [6, 7, 8, 9]. The central idea behind sGDML is to utilize a kernel function, denoted as $\mathbf{k}(\mathbf{x}, \mathbf{x}') = \nabla_{\mathbf{x}} k_E(\mathbf{x}, \mathbf{x}') \nabla_{\mathbf{x}'}^{\top}$, which models the force field $\mathbf{f}_{\mathbf{F}}$ as a transformation of an unknown potential energy surface f_E. Specifically, we have:

$$\mathbf{f}_{\mathbf{F}} = -\nabla f_E \sim \mathcal{GP}\left[-\nabla \mu_E(\mathbf{x}), \nabla_{\mathbf{x}} k_E(\mathbf{x}, \mathbf{x}') \nabla_{\mathbf{x}'}^{\top}\right]. \quad (1)$$

where $\mu_E : \mathbb{R}^d \to \mathbb{R}$ and $k_E : \mathbb{R}^d \times \mathbb{R}^d \to \mathbb{R}$ are the prior mean and covariance functions, respectively, that define the latent energy-based Gaussian process. The chemical structure descriptor, denoted by $\mathbf{x} \in \mathbb{R}^d$, is constructed from the pairwise inverse distances (d), ensuring the rototranslational invariance of the energy. Symmetries are recovered using a data-driven multipartite matching approach, which automatically identifies permutations of atoms present in the training set.

sGDML is capable of modeling systems with hundreds of atoms without imposing localization constraints [8]. Despite its non-parametric nature, sGDML force fields typically utilize around one order of magnitude fewer parameters than deep neural network architectures, resulting in less costly computational evaluations.

Models employed in the current study were trained using readily available sGDML code (version 1.0.2) with default settings (cf. [9] and github.com/stefanch/sGDML). Hyperparameter search of σ was performed from 10 to 200 with a step of 10. Models for Challenge I were trained using the analytic solver, while models for Challenges II and III employed the iterative solver.

1.4 FCHL19*

The model used in our work for learning energies and forces utilizes a form of sparse Gaussian Process regression[10, 11, 12, 13] (GPR) known as Operator Quantum Machine Learning (OQML).[14, 15] The energies ($U\mathbf{U}$) and forces ($F\mathbf{F}$) on query atoms are obtained as

$$\mathbf{U} = \mathbf{K}^{\mathrm{OQML}}\boldsymbol{\alpha}^{\mathrm{OQML}}$$
$$\mathbf{F} = -\frac{\partial}{\partial \mathbf{r}}\mathbf{K}^{\mathrm{OQML}}\boldsymbol{\alpha}^{\mathrm{OQML}} \tag{2}$$

where $\mathbf{r}$ denotes coordinates of the query atom and the elements of the non-square kernel matrix $\mathbf{K}^{\mathrm{OQML}}$ are given by

$$\mathbf{K}^{\mathrm{OQML}}_{iJ} = \sum_{I \epsilon i} \mathcal{K}(\mathbf{q}_J, \mathbf{q}_I) \tag{3}$$

with $\mathcal{K}$ being a suitable kernel function (gaussian in our work), $\mathbf{q}$ denoting atomic representation feature vectors, J denoting an atom in the training set and I is an atom in the query molecule i. The regression coefficients $\boldsymbol{\alpha}^{\mathrm{OQML}}$ are obtained by solving the following equation via singular value decomposition during training

$$\begin{pmatrix} \mathbf{U}^{\mathrm{train}} \\ \mathbf{F}^{\mathrm{train}} \end{pmatrix} = \begin{pmatrix} \mathbf{K}^{\mathrm{OQML}} \\ -\frac{\partial}{\partial \mathbf{r}}\mathbf{K}^{\mathrm{OQML}} \end{pmatrix} \boldsymbol{\alpha}^{\mathrm{OQML}} \tag{4}$$

For fast training and prediction we used the recently introduced graphics processing unit (GPU)-accelerated implementation of approximate kernel methods called Quantum Machine Learning (QML)-Lightning which can provide energy and force predictions on a microsecond per atom timescale. QML-Lightning relies on approximating the kernel function $\mathcal{K}(\mathbf{q}_J, \mathbf{q}_I)$ with a lower-dimensional mapping using Random Fourier Features[16] (RFF)

$$\mathcal{K}(\mathbf{q}_J, \mathbf{q}_I) = \langle \phi(\mathbf{q}_J), \phi(\mathbf{q}_I) \rangle \approx \mathbf{z}(\mathbf{q}_J)^T \mathbf{z}(\mathbf{q}_I) = \frac{1}{N_f}\sum_{i=1}^{N_f} e^{-i\mathbf{w}_i^T(\mathbf{q}_J - \mathbf{q}_I)} \tag{5}$$
$$\mathbf{z}(\mathbf{q}_I) = \cos(\mathbf{W}[\mathbf{q}_I, \mathbf{1}]^T)$$

where N_f is the number of independent vectors $\mathbf{w}$ drawn from the probability distribution $p(\mathbf{w})$ (which is a Gaussian for a Gaussian kernel) and $\mathbf{W}[\mathbf{q}_I, \mathbf{1}]^T$ is an affine transformation. The matrix $\mathbf{W}$ can be efficiently computed and stored using structured orthogonal random features (SORF)[17] which replaces it with products of random binary diagonal matrices ($\mathbf{D}$) and Walsh-Hadamard matrices ($\mathbf{H}$)

$$\mathbf{W}_{\mathrm{SORF}} \approx \frac{\sqrt{d}}{\sigma}\left[\mathbf{HD}_1, ..., \mathbf{HD}_{\frac{N_f}{d}}\right] \tag{6}$$

with d denoting the dimension of $\mathbf{q}$ and σ is a hyperparameter of the kernel. The feature vectors $\mathbf{q}$ are projected onto 2^n dimensions via principal component analysis (PCA) in order to be consistent with the Walsh-Hadamard matrices. In our work $N_{\mathrm{PCA}} = 128$.
The atomic representation feature vector employed alongside QML-Lightning in our work is the atom-centered FCHL19[18] representation which uses discretized bins of weighted two- and three- body distributions.

1.5 SOAP/GAP

1.5.1 Gaussian Approximation Potentials

Gaussian Approximation Potentials (GAPs) are a widely used class of ML potentials based on sparse Gaussian Process Regression. The GAP approach has been described extensively in the literature[19, 20, 21, 12]. Here, we provide a brief overview for completeness.

The GAP models used herein are based on a combination of two-body (2B) and many-body (MB) kernels. The former use interatomic distances (r_{ij}) as descriptors and have been found to improve the stability of the potentials (e.g. by avoiding close contacts between atoms).[22] For the MB kernels, the Smooth Overlap of Atomic Positions (SOAP)[20] descriptor is used, which provides a rotationally invariant fingerprint of the local atomic environment of an atom ($\boldsymbol{\xi}_i$), based on spherical harmonics and radial basis functions with a given cutoff r_{cut}.

The GAP energy for a given configuration can then be described as:

$$E_{\mathrm{GAP}} = \sum_{i,j} (\delta_{\mathrm{2B}})^2 \sum_{m=1}^{M^{\mathrm{2B}}_{\mathrm{sparse}}} c_{\mathrm{2B},m} k_{\mathrm{2B}}(r_{ij}, r_m) + \sum_i (\delta_{\mathrm{MB}})^2 \sum_{m=1}^{M^{\mathrm{MB}}_{\mathrm{sparse}}} c_{\mathrm{MB},m} k_{\mathrm{MB}}(\boldsymbol{\xi}_i, \boldsymbol{\xi}_m) \tag{7}$$

Here, the total energy in Eq. 7 is composed of 2B and MB terms, with the contributions from each weighted by δ_{2B} and δ_{MB}, respectively. The individual energy contributions in turn consist of trainable regression coefficients c and kernel functions k, detailed below. The summation for the 2B term is performed for all atom pairs i, j within a predefined cutoff, while the MB energy accumulates over each atom i. Owing to the sparsification in GAPs, the kernel functions for both

the 2B and MB components are evaluated only on subsets of M representative points, which are usually significantly smaller than the total number of training data points. This dramatically decreases the computational costs associated with training and predictions and breaks the unfavorable scaling of these steps with the size of the training set. However, it naturally implies that a judicious selection of these sparse points is crucial. Herein, sparse points are sampled uniformly for the 2B term and selected via CUR decomposition for the MB term.

In the current work, the Gaussian kernel is used as a 2B kernel, and a fourth-order polynomial kernel ($\zeta = 4$) is used for the MB kernels, as shown in Eq. 8 and 9:

$$k_{2\mathrm{B}}(r_{ij}, r_m) = \exp\left(-\frac{\|r_{ij} - r_m\|^2}{2\sigma_{2\mathrm{B}}^2}\right) \tag{8}$$

and

$$k_{\mathrm{MB}}(\boldsymbol{\xi}_i, \boldsymbol{\xi}_m) = (\boldsymbol{\xi}_i, \boldsymbol{\xi}_m)^{\zeta} \tag{9}$$

Note that in general multiple MB kernels can be combined, often using a high-resolution, small cutoff descriptor and a low-resolution, large cutoff descriptor. This is also the case herein.

1.5.2 Hyperparameter selection

Hyperparameters	Two-body	Many-body	Unit
M	20	2000	
n_{max}	-	8	
l_{max}	-	4	
σ_{E}	0.01	0.01	kcal/mol
σ_{F}	6.7×10^{-4}	6.7×10^{-4}	kcal/(mol·Å)

Table SI 1: GAP hyperparameters used in challenge 1 (Ac-Ala3-NHMe) for the largest training set. The values of σ_{E} and σ_{F} were reoptimized for smaller training set sizes.

Hyperparameters	Two-body	Many-body	Unit
Complete			
M	25	2000	
n_{max}	-	8	
l_{max}	-	4	
σ_{E}	0.01	0.01	kcal/mol
σ_{F}	5×10^{-4}	$\times10^{-4}$	kcal/(mol·Å)
Incomplete			
M	25	2000	
n_{max}	-	6	
l_{max}	-	3	
σ_{E}	0.01	0.01	kcal/mol
σ_{F}	6.7×10^{-4}	6.7×10^{-4}	kcal/(mol·Å)

Table SI 2: GAP hyperparameters used in challenge 2 (AcPheAla5Lys peptide) for the largest training set. The values of σ_{E} and σ_{F} were reoptimized for smaller training set sizes.

Hyperparameters	Two-body	Many-body	Unit
M	20	2000	
n_{max}	-	8	
l_{max}	-	4	
σ_{E}	0.001	0.001	kcal/mol
σ_{F}	5×10^{-5}	5×10^{-5}	kcal/(mol·Å)

Table SI 3: GAP hyperparameters used in challenge 3 (1-8-naphthyridine on graphene) for the largest training set. The values of σ_{E} and σ_{F} were reoptimized for smaller training set sizes.

For good model performance, the various hyperparameters described above should be set to reasonable values. Fortunately, robust heuristics have been developed for many SOAP and GAP hyperparameters. Specifically, `Universal SOAP` descriptors have been defined for the full periodic table in Ref. [23], which facilitates the straightforward setting of critical hyperparameters such as r_{cut}, $\sigma_{2\mathrm{B}}$, σ_{MB}, etc. Additionally, the `wfl` package provides a multi-stage fitting scheme, which automatically determines the kernel weighting hyperparameters δ[24].

Hyperparameters	Two-body	Many-body	Unit
	Unfolded		
M	20	2500	
n_{max}	-	6	
l_{max}	-	3	
σ_{E}	0.1	0.1	kcal/mol
σ_{F}	0.002	0.002	kcal/(mol·Å)

Table SI 4: GAP hyperparameters used in challenge 4 (MAPbI) for the largest training set. The values of σ_{E} and σ_{F} were reoptimized for smaller training set sizes.

This leaves a small number of remaining hyperparameters, which can be efficiently determined using limited low-dimensional grid searches. These are the size of the representative sets used for sparsification (M), the number of radial and angular basis functions for the SOAP descriptor (n_{max} and l_{max}), and the regularization parameters (σ_{E} and σ_{F}) that define target accuracies for energies and forces, in order to avoid overfitting. These are adjusted for each challenge. The final choices of hyperparameters are presented in Tabs. SI 1–SI 4.

1.6 PhysNet

PhysNet participated in the TEA Challenge 2023.

PhysNet is a 'message-passing' neural network designed to predict atomic properties[25, 26]. It uses learnable descriptors of atomic environments to estimate the energy contributions (E_i) and partial charges (q_i) for individual atoms. Initially, these descriptors are set as $\boldsymbol{x}_i^0 = \mathbf{e}_{Z_i}$, where $\mathbf{e}_{Z_i}$ represents a parameter vector based on the nuclear charge Z_i initialized randomly to values between $[-\sqrt{3}\ldots\sqrt{3}]$. These descriptors are then iteratively refined through message-passing between atoms within a specified cutoff distance (r_{cut}). The trained descriptors are then used to predict the total energy of the chemical system by summation of the atomic contributions and explicitly including long-range electrostatics and dispersion according to

$$E = \sum_i E_i + k_e \sum_{i=1}^{N_{\mathrm{atom}}} \sum_{j>i}^{N_{\mathrm{atom}}} \frac{q_i q_j}{r_{ij}} + E_{\mathrm{D}_3} \tag{10}$$

and the partial charges q_i (which are corrected to ensure total charge conservation). Here, k_e represents Coulomb's constant and the second term involving $\frac{q_i q_j}{r_{ij}}$ is damped to avoid numerical instabilities caused by the singularity at $r_{ij} = 0$ (for details refer to Ref. [26]). The forces $\boldsymbol{F}$ can be obtained using reverse mode automatic differentiation[27] as implemented in Tensorflow[28]. All hyperparameters used in the present work are given in Table SI 5. Various combinations of hyperparameters for the models were tested, but the results did not differ significantly.

Table SI 5: Hyperparameters of all models used in this work

Hyperparameter	Value	Significance
F	128	Dimensionality of feature space
K	64	Number of radial basis functions
N_{module}	5	Number of stacked modular building blocks
$N_{\text{residual}}^{\text{atomic}}$	2	Number of residual blocks for atom-wise refinements
$N_{\text{residual}}^{\text{interaction}}$	3	Number of residual blocks for refinements of proto-message
$N_{\text{residual}}^{\text{output}}$	1	Number of residual blocks in output blocks
r_{cut}	10 Å	Cutoff radius for interactions in the neural network

PhysNet models were only trained for the complete data set containing folded and unfolded structures of Ac-Ala3-NHMe (*Challenge 1*). The training was performed for training/validation set sizes of [200, 400, 600, 800, 1000] and, for each data set size, two models were trained on different splits of the data (although keeping training and validation data fixed.). After initialization, the parameters of PhysNet are optimized using AMSGrad[29] with default parameters and a learning rate of 10^{-3}. The training was performed with a batch size of 73 and the relative contribution of the force term is weighted roughly 50 times more than the energies. During training, an exponential moving average of all parameter values is kept using a decay rate of 0.999. Overfitting is prevented using early stopping. Note that the energies have been standardized by subtracting the mean energy of the data. The results are reported for the model with lower MAEs/RMSEs.

All MD simulations which were run for the determination of R_g were carried out using the atomic simulation environment (ASE)[30]. These were run in the NVT ensemble at 300 K using a Langevin thermostat with a friction coefficient of 10^{-3}, a time step of 0.5 fs and using the model trained on 1000 training points. The radius of gyration, R_g, is calculated as

$$R_g = \sqrt{\frac{\sum_{i=1}^{N} m_i \left(\boldsymbol{r}_i - \boldsymbol{r}_{\text{CM}}\right)^2}{\sum_{i=1}^{N} m_i}} \tag{11}$$

with all hydrogen atoms excluded.

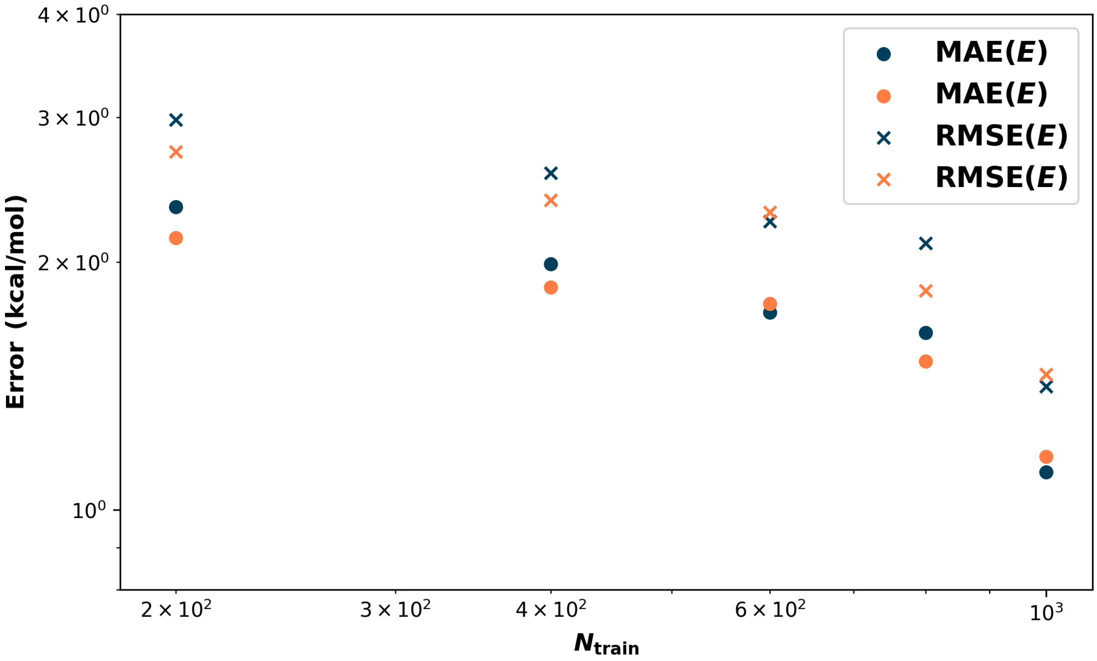

Fig. SI 1: Energy learning curves for all data set sizes as obtained on the test data set. Two models are trained on different splits of the train/validation data each.

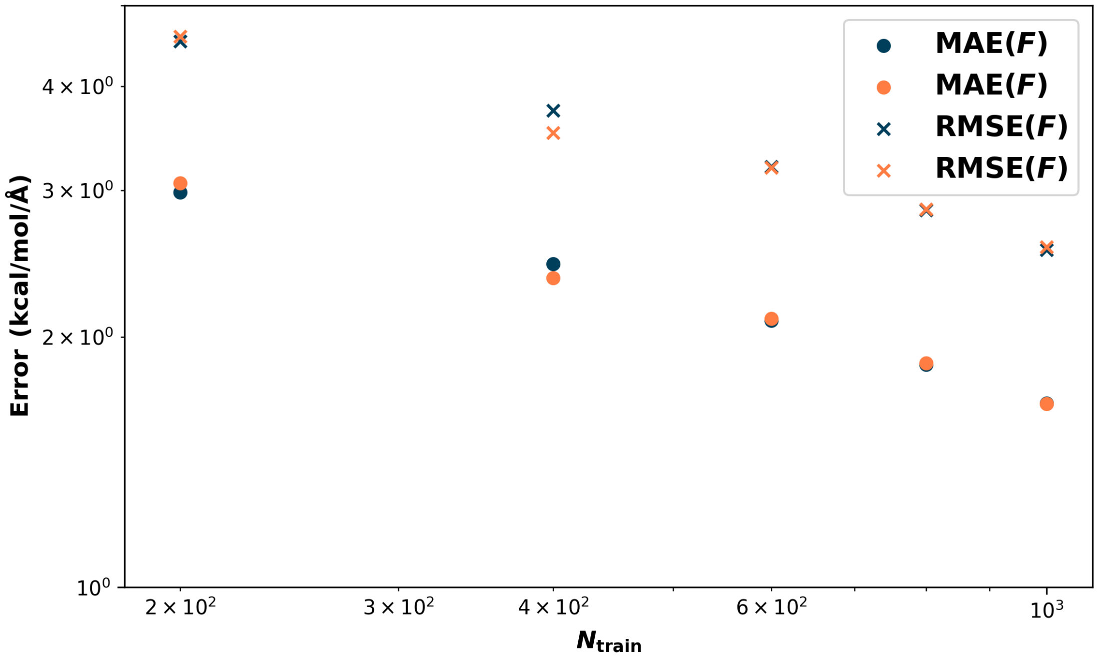

Fig. SI 2: Force learning curves for all data set sizes as obtained on the test data set. Two models are trained on different splits of the train/validation data each.

$N_{\text{train}}/N_{\text{valid}}$	200		400		600		800		1000	
	Valid	Test	Valid	Test	Valid	Test	Valid	Test	Valid	Test
MAE(E)	2.11	2.14	1.93	1.86	1.77	1.74	1.43	1.52	1.13	1.11
RMSE(E)	2.70	2.73	2.41	2.38	2.27	2.24	1.78	1.85	1.45	1.41
MAE(F)	3.04	3.06	2.35	2.35	2.05	2.09	1.86	1.86	1.67	1.66
RMSE(F)	4.51	4.59	3.49	3.52	3.13	3.20	2.86	2.85	2.61	2.55

Table SI 6: MAEs and RMSEs on the validation and test (20000 samples) data sets for PhysNet. Errors on energies are given in kcal/mol while errors on forces are given in kcal/mol/Å

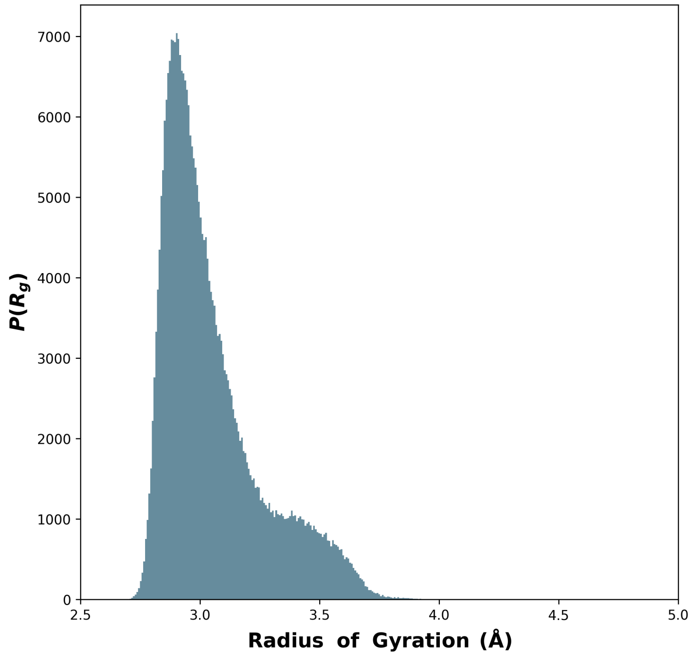

Fig. SI 3: Radius of gyration, R_g for a PhysNet model trained on 1000 training structures of the complete data set (i.e. containing folded and unfolded structures). Note that the PES was not robust for all the trajectories and the distribution of R_g was obtained from stable trajectories only. Here, R_g was obtained from an aggregate of ~ 31.2 ns.

2 Warm dense Hydrogen benchmark

The following summarises a presentation given at the TEA Challenge (Crash TEsting machine learning force fields: Applicability, best practices, limitations) workshop on October 23, 2023, at the University of Luxembourg. Details can be found in Thomas Bischoff, Bastian Jäckl, Matthias Rupp: *Hydrogen under Pressure as a Benchmark for Machine-Learning Potentials*[31].

Machine-learning potentials (MLPs) can accelerate ab-initio molecular dynamics simulations by several orders of magnitude. [32] Their performance is commonly measured as the prediction error in energies and forces on data not used for training. While low prediction errors on a test set are necessary, they do not guarantee good performance in dynamics simulations. Even in situations where test-set errors do correlate strongly with simulation performance, it is unclear from the test-set errors alone if simulation performance will meet expectations.

Evaluating the performance of MLP-accelerated simulations requires physically motivated performance measures obtained via running MLP-accelerated simulations. The adoption of such measures, however, has been limited by the effort and domain knowledge required to calculate and interpret them. Overcoming this limitation requires benchmark automatisation, including benchmark execution and analysis of results.

For this purpose, we created data and scripts to automatically quantify the performance of MLPs in dynamics simulations of warm dense hydrogen. For this challenging benchmark system, we provide geometries, energies, forces, and stresses, calculated at the density functional level of theory for different temperatures and mass densities. We also provide scripts to automatically calculate, quantitatively compare, and visualize pressures, diffusion coefficients, radial distribution functions, and stable molecular fractions as functions of cell density.

Employing this benchmark, we show that several state-of-the-art MLPs fail to reproduce a crucial liquid-liquid phase transition. Specifically, we tested the (reparametrised) Yukawa and Tersoff classical empirical potentials, as well as ultrafast potentials, [32] PACE, and MACE. Only the MACE model was able to capture the physics of the phase transition correctly.

3 Atomic Force MAEs for Challenge I

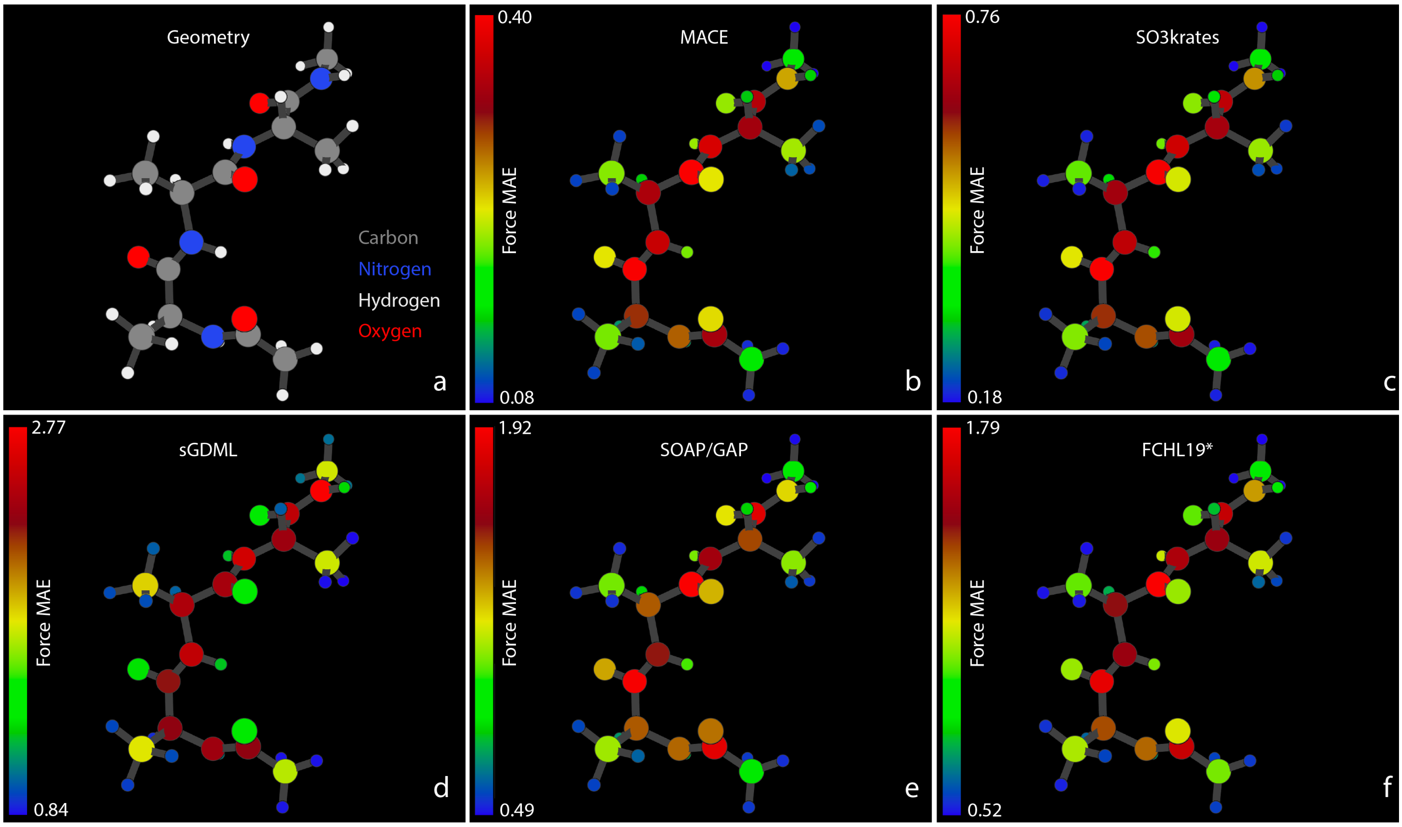

Fig. SI 4: Atomic Force MAEs for Ac-Ala3-NHMe. Fig. (a) shows a snapshot of the system geometry and atom types: Carbons – grey, Nitrogens – blue, Hydrogens – white, and Oxygens – red. Figures (b) through (f) display the MAEs for forces, measured in kcal/(mol·Å), acting on individual atoms within the Ac-Ala3-NHMe. The MAEs correspond to the MLFFs predictions on the test set and are represented with different colors according to the color bars shown with the corresponding scaling numbers, different for different MLFFs. Note the rather different absolute scale of the colour bars, ranging from 0.40 for MACE to 2.77 for sGDML.

4 Maximum force prediction errors

Figures SI 4, SI 5, and SI 6 illustrate the color-coded force prediction atomic errors for Challenges I, II, and III, respectively, highlighting the scenarios where the MLFFs deliver the least accurate predictions. The same system geometries, with atoms colored according to their chemical elements, are displayed adjacent to the absolute force error plots. Please note that the error scales differ between each figure.

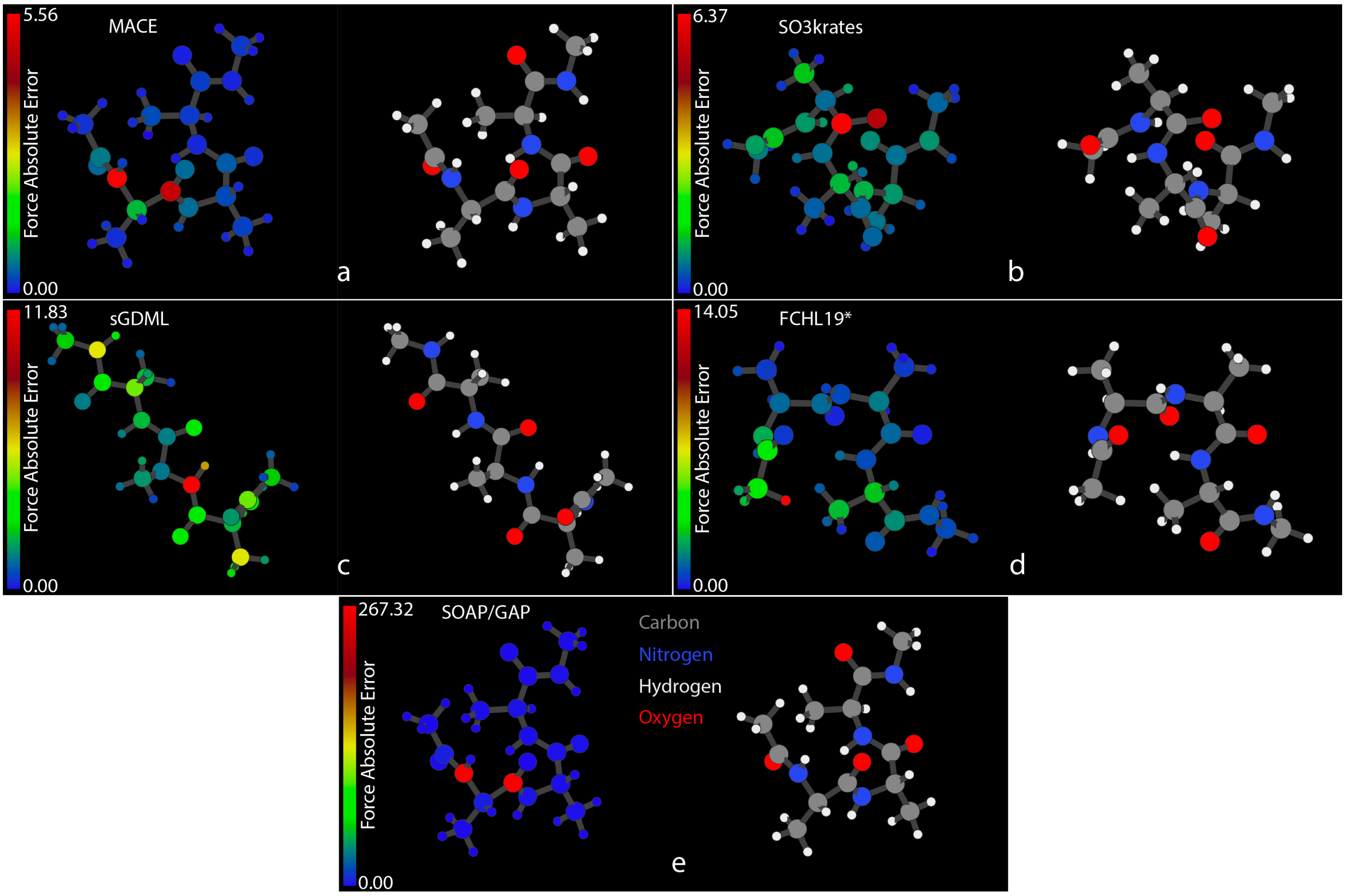

Fig. SI 5: Maximum Atomic Force Errors for Ac-Ala3-NHMe. Figures (a) through (e) display the absolute atomic force errors, measured in kcal/(mol·Å), acting on individual atoms within the Ac-Ala3-NHMe system for the geometries where these errors are maximum for the corresponding ML model. The MAX values are represented with different colors according to the color bars shown with the scaling numbers, different for different MLFFs.

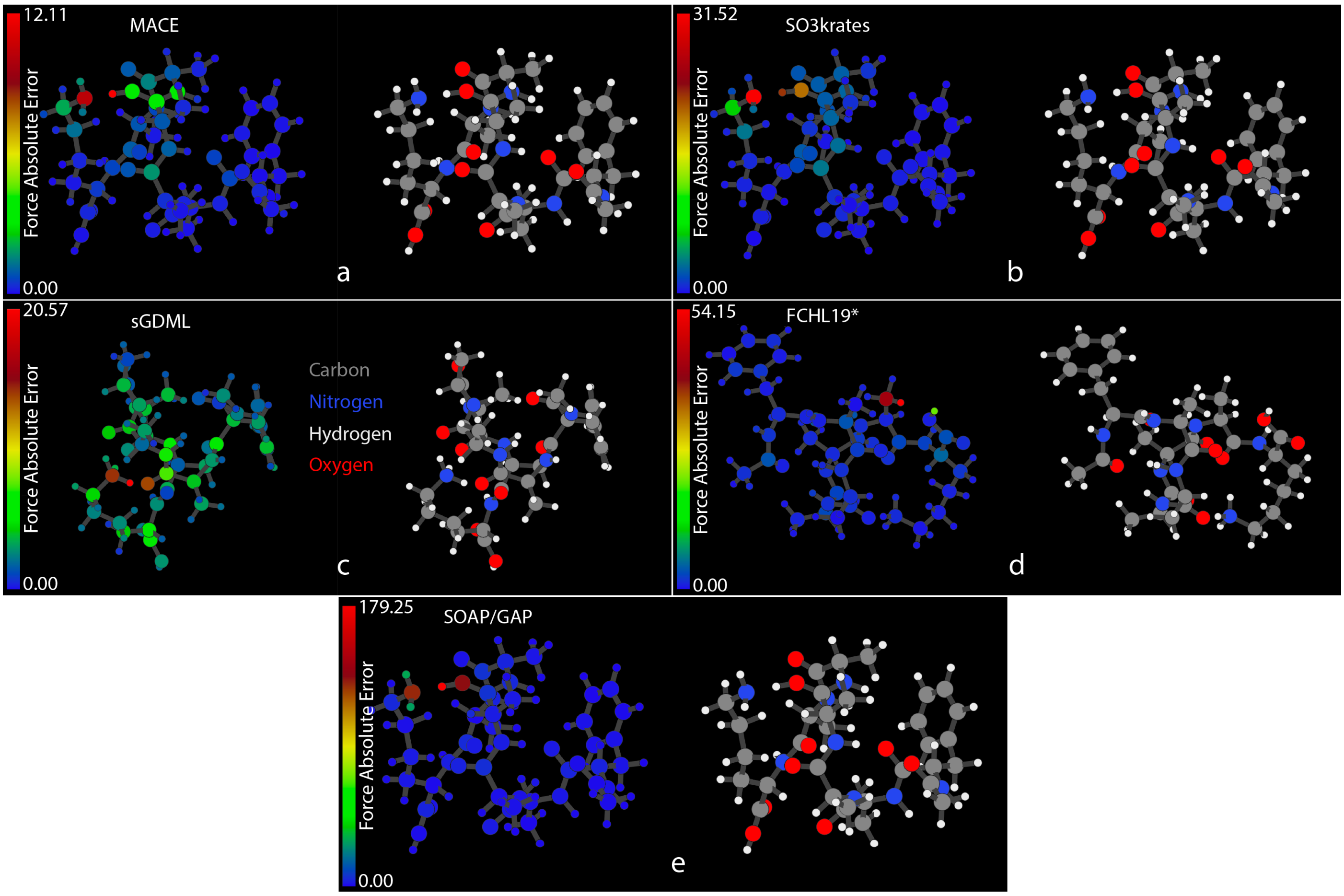

Fig. SI 6: Maximum Atomic Force Errors for Ac-Phe-Ala5-Lys. Figures (a) through (e) display the absolute atomic force errors, measured in kcal/(mol·Å), acting on individual atoms within the Ac-Phe-Ala5-Lys system for the geometries where these errors are maximum for the corresponding ML model. The MAX values are represented with different colors according to the color bars shown with the scaling numbers, different for different MLFFs.

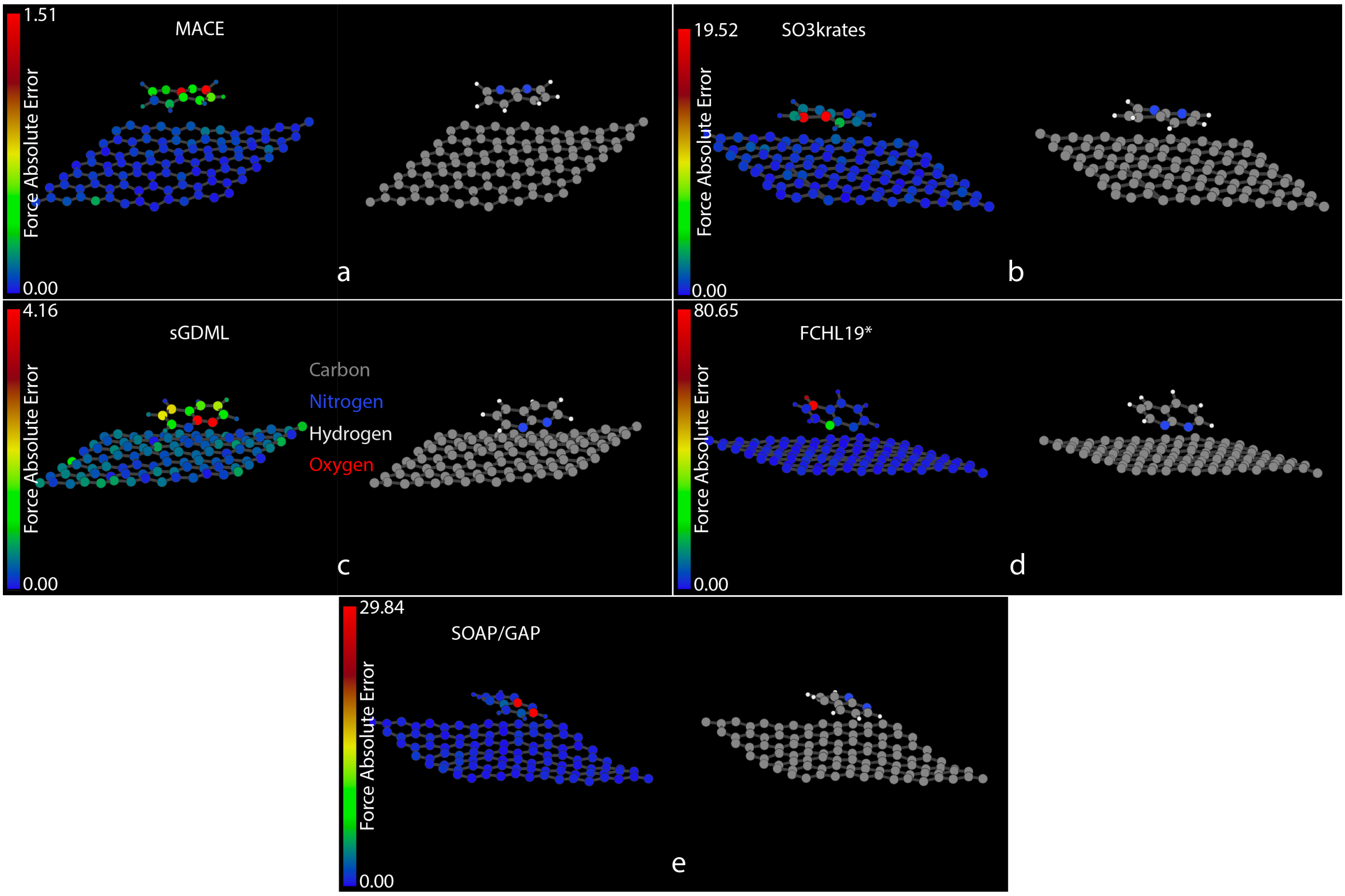

Fig. SI 7: Maximum Atomic Force Errors for $C_8H_6N_2/C_{98}$. Figures (a) through (e) display the absolute atomic force errors, measured in kcal/(mol·Å), acting on individual atoms within the $C_8H_6N_2/C_{98}$ system for the geometries where these errors are maximum for the corresponding ML model. The MAX values are represented with different colors according to the color bars shown with the scaling numbers, different for different MLFFs.

5 TASK3: Retrained SO3krates model

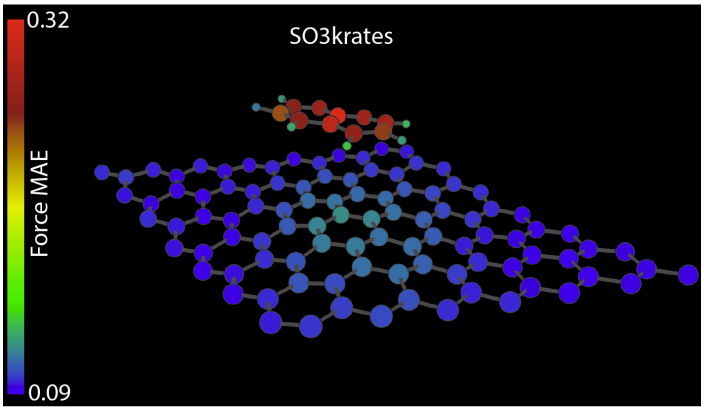

Fig. SI 8: Atomic Force MAEs for $C_8H_6N_2/C_{98}$. The MAEs in kcal/(mol·Å) correspond to the retrained SO3krates model predictions on the test set and are represented in different colors according to the colour bar shown with the corresponding scaling numbers.

The significant errors for C atoms on the two borders for SO3krates, observed in Fig. 6, are due to the JAX default of running in tensorflow32 precision on A100 and H100 GPU. This problem can be alleviated by explicitly disabling tensorflow32 precision (which performs some operations in float16) and instead running training and evaluation in standard float32 precision. This not only alleviates the boundary effects, as shown in Fig. SI 7, but also gives significantly lower errors, ranging from 0.09 to 0.32 kcal/(mol·Å)

6 Normalized errors on the test sets

Challenge		MACE			SO3			sGDML			SOAP/GAP			FCHL19*		
I (com)	E	0.54	0.68	3.88	1.20	1.52	7.11	3.87	5.06	27.3	4.14	5.34	32.1	2.70	3.48	16
	F	1.01	1.54	53.3	1.99	2.93	91.0	7.77	11.0	143	5.20	8.65	2580	4.99	7.03	152
	F(H)	0.80	1.18	64.1	1.67	2.36	82.8	7.07	9.40	155	4.61	6.22	160	4.61	6.30	209
	F(C)	1.07	1.49	33.6	2.07	2.81	65.2	7.88	10.4	95.1	5.23	8.18	1850	5.04	6.67	82
	F(N)	1.24	1.69	39.6	2.38	3.17	39.9	9.85	13.0	106	5.61	10.6	1890	5.46	7.09	64
	F(O)	1.23	1.71	49.8	2.37	3.18	76.0	7.33	9.87	118	6.88	8.96	97.4	5.62	7.43	106
I (fold-fold)	E	0.28	0.36	2.01	0.91	1.14	5.13	2.26	3.00	28.6	2.52	3.25	15.9	1.67	2.15	12
	F	0.99	1.51	48.6	1.96	2.87	113	7.41	10.6	148	5.05	7.20	435	4.80	6.74	88
	F(H)	0.80	1.18	66.5	1.69	2.40	154	6.74	8.99	133	4.50	6.09	175	4.45	6.04	121
	F(C)	1.04	1.45	30.0	2.02	2.72	36.9	7.58	10.1	105	5.10	6.85	309	4.85	6.39	60
	F(N)	1.22	1.68	34.8	2.35	3.13	49.2	9.26	12.3	111	5.34	7.10	326	5.32	6.91	63
	F(O)	1.22	1.70	46.2	2.30	3.09	57.1	6.86	9.35	116	6.70	8.78	139	5.36	7.06	69
I (fold-unfold)	E	0.79	1.02	4.80	2.34	2.79	10.3	18.4	26.1	112	5.34	6.67	25.9	6.39	8.33	31.7
	F	1.33	2.08	57.4	2.52	3.79	60.7	13.6	19.7	207	5.91	8.47	114	5.88	8.51	108
	F(H)	1.00	1.47	80.4	2.07	2.99	85.1	12.0	16.5	244	5.19	7.17	159	5.30	7.40	121
	F(C)	1.45	2.05	29.5	2.59	3.54	30.1	14.1	18.9	140	5.92	7.90	69.6	6.04	8.13	77
	F(N)	1.60	2.16	26.1	3.10	4.21	34.8	16.6	21.7	151	6.44	8.43	80.7	6.54	8.62	68
	F(O)	1.77	2.39	28.7	3.18	4.32	35.8	13.2	18.1	147	7.96	10.4	111	6.63	8.80	83
I (unfold-unfold)	E	0.84	0.98	3.36	1.14	1.43	6.37	3.20	5.31	92.7	3.35	4.33	16.2	2.14	3.01	31.6
	F	0.84	1.33	45.7	1.66	2.45	60.8	6.38	9.37	231	4.76	6.67	109	4.33	6.06	98
	F(H)	0.72	1.09	55.5	1.48	2.07	61.6	6.07	8.36	323	4.42	5.92	121	4.24	5.80	132
	F(C)	0.87	1.26	20.0	1.68	2.32	43.4	6.35	8.74	109	4.78	6.31	56.1	4.24	5.59	47
	F(N)	1.00	1.45	33.4	1.93	2.63	36.6	7.79	10.6	119	4.88	6.35	79.5	4.62	6.08	71
	F(O)	0.98	1.40	28.2	1.86	2.52	34.1	5.76	8.02	88.5	5.90	7.66	56.5	4.66	6.12	67
I (unfold-fold)	E	2.08	2.70	11.3	1.95	2.36	10.2	53.1	70.7	319	4.40	5.62	22.6	9.17	12.9	75
	F	2.08	3.82	131	2.93	4.52	113	22.9	34.5	468	6.44	9.59	503	8.83	13.6	353
	F(H)	1.39	2.45	180	2.32	3.67	154	20.9	32.7	641	5.67	8.67	689	8.39	13.7	484
	F(C)	2.19	3.69	53.3	3.03	4.21	73.0	22.3	30.5	278	6.14	8.34	194	7.85	10.9	162
	F(N)	2.91	4.15	96.4	3.73	4.96	56.2	28.5	37.8	236	7.62	10.2	221	11.8	16.2	151
	F(O)	3.37	5.27	69.2	3.92	5.42	104	26.4	37.8	314	9.33	12.8	180	10.9	15.5	294
II (comp)	E	0.35	0.45	4.97	0.80	1.00	4.19	6.12	8.40	43.9	12.8	16.0	69.6	3.94	4.99	23
	F	0.59	0.88	170	1.57	2.37	455	7.19	10.6	280	25.5	35.1	1910	8.09	11.3	621
	F(H)	0.52	0.75	238	1.40	2.04	409	6.69	9.45	393	24.7	33.5	1630	7.59	10.2	871
	F(C)	0.60	0.83	37.7	1.59	2.22	62.0	7.19	9.88	137	26.6	34.4	404	8.37	11.0	305
	F(N)	0.68	0.93	90.4	1.84	2.54	333	9.08	12.4	176	23.6	30.4	879	8.10	10.4	222
	F(O)	0.74	1.03	115	1.89	2.61	221	6.97	9.85	192	25.5	33.4	1930	8.77	11.5	183
II (incom-incom)	E	0.36	0.51	20.6	0.65	0.83	14.4	7.90	11.3	60.7	13.8	17.2	60.3	3.87	4.88	24
	F	0.53	0.85	420	1.49	2.28	442	6.31	9.55	279	26.1	35.8	2170	8.06	11.2	636
	F(H)	0.48	0.86	587	1.35	2.06	617	5.85	8.42	390	25.1	33.9	2420	7.54	10.2	889
	F(C)	0.53	0.74	65.6	1.50	2.11	137	6.31	8.89	119	27.6	35.4	389	8.31	10.9	317
	F(N)	0.61	0.90	129	1.69	2.40	196	7.96	11.1	120	24.1	31.2	1590	8.10	10.4	224
	F(O)	0.65	0.97	182	1.79	2.57	412	6.17	8.98	256	25.0	32.2	490	8.88	11.7	555
II (incom-unkn)	E	0.44	0.56	2.59	0.92	1.17	6.31	60.9	71.6	346	16.2	20.3	82.3	5.33	6.81	35
	F	0.70	1.07	80.4	1.83	2.82	173	13.1	18.5	497	25.9	35.7	1060	8.29	11.6	263
	F(H)	0.57	0.85	75.0	1.53	2.33	245	13.7	19.9	702	25.2	34.1	1130	7.78	10.6	293
	F(C)	0.73	1.01	28.1	1.90	2.69	65.2	12.1	15.9	154	27.4	35.2	395	8.52	11.2	156
	F(N)	0.85	1.12	34.1	2.19	2.95	78.6	15.4	20.0	167	23.9	30.9	774	8.44	10.9	175
	F(O)	0.92	1.28	79.7	2.29	3.18	123	13.3	17.8	201	24.3	31.4	397	9.00	11.9	261
III	E	0.14	0.18	0.73	0.24	0.30	1.81	0.26	0.33	1.54	0.75	0.92	2.83	211	211	219
	F	0.21	0.34	18.8	2.48	4.12	194	1.00	1.66	54.2	2.51	3.36	308	6.39	8.46	842
	F(H)	0.42	0.58	14.9	1.87	2.66	57.4	1.19	1.72	18.4	2.92	4.18	165	8.59	11.4	892
	F(C)	0.19	0.31	18.5	2.51	4.16	191	0.98	1.62	50.0	2.47	3.29	303	6.26	8.26	828
	F(N)	0.52	0.76	10.7	2.05	2.90	51.6	1.74	2.71	47.1	3.57	4.73	80.9	8.52	11.2	348
	F(mol)	5.90	7.90	67.0	28.3	37.8	243	8.03	12.0	130	76.6	128	965	197	299	2011
IV	E	0.45	0.58	1.93	0.68	0.85	2.75	-	-	-	1.49	1.88	6.28	102	106	192
	F	1.56	2.14	45.3	1.87	2.63	36.9	-	-	-	4.00	5.50	66.9	26.4	34.0	642
	F(H)	1.10	1.47	43.6	1.19	1.57	22.3	-	-	-	2.63	3.46	64.4	24.8	32.1	618
	F(C)	0.85	1.12	19.2	0.91	1.17	18.6	-	-	-	2.39	3.04	32.7	12.3	16.2	322
	F(N)	0.96	1.24	11.5	1.03	1.31	8.10	-	-	-	2.44	3.09	22.9	13.4	17.3	283
	F(Pb)	4.80	6.17	55.1	7.02	8.90	44.1	-	-	-	14.3	18.0	114	58.4	73.9	589
	F(I)	4.72	6.22	86.2	6.18	8.16	60.6	-	-	-	11.9	15.5	118	66.2	84.1	799
	F(mol)	6.20	8.03	102	6.12	7.77	63.0	-	-	-	15.0	19.1	141	171	217	1449

Table SI 7: MAE, RMSE, and MAX errors for relative energy and forces are reported in %, w.r.t. the mean absolute energies and forces in the reference DFT datasets.

Challenge		MACE			SO3			sGDML			SOAP/GAP			FCHL19*		
I (com)	E	1.86	2.35	13.5	4.16	5.26	24.6	13.4	17.5	94.7	14.3	18.5	111	9.35	12.0	56.3
	F	0.75	1.14	39.5	1.47	2.17	67.5	5.77	8.22	106	3.86	6.41	1920	3.70	5.21	112
	F(H)	0.61	0.89	48.5	1.27	1.79	62.7	5.36	7.12	117	3.49	4.71	121	3.49	4.77	158
	F(C)	0.85	1.18	26.6	1.64	2.22	51.6	6.24	8.25	75.3	4.14	6.48	1460	3.99	5.28	65.0
	F(N)	0.97	1.31	30.8	1.85	2.47	31.0	7.67	10.1	82.7	4.36	8.28	1470	4.25	5.52	50.3
	F(O)	0.92	1.27	37.2	1.77	2.37	56.7	5.47	7.36	87.9	5.13	6.68	72.7	4.19	5.54	79.4
I (fold-fold)	E	1.56	2.00	11.1	5.06	6.29	28.4	12.5	16.6	158	14.0	18.0	88.3	9.26	11.9	69.4
	F	0.73	1.12	36.0	1.45	2.13	83.7	5.50	7.88	110	3.75	5.34	323	3.56	5.00	65.7
	F(H)	0.60	0.89	50.2	1.27	1.81	117	5.09	6.79	101	3.40	4.60	132	3.36	4.56	91.5
	F(C)	0.82	1.15	23.8	1.60	2.16	29.2	6.00	8.00	83.5	4.04	5.42	245	3.84	5.06	48.2
	F(N)	0.95	1.30	27.1	1.82	2.43	38.3	7.20	9.58	86.3	4.15	5.52	253	4.13	5.37	49.4
	F(O)	0.91	1.27	34.5	1.72	2.31	42.7	5.12	6.99	86.6	5.00	6.56	104	4.00	5.28	51.5
I (fold-unfold)	E	2.58	3.37	15.8	7.70	9.19	33.9	60.6	85.7	368	17.5	21.9	85.0	21.0	27.4	104
	F	0.99	1.54	42.5	1.87	2.81	45.0	10.1	14.6	154	4.38	6.28	84.3	4.36	6.31	80.1
	F(H)	0.77	1.12	61.3	1.58	2.28	64.9	9.19	12.6	186	3.96	5.47	122	4.04	5.65	92.4
	F(C)	1.15	1.62	23.3	2.05	2.80	23.8	11.1	15.0	111	4.69	6.26	55.1	4.78	6.44	61.1
	F(N)	1.25	1.68	20.3	2.42	3.28	27.2	12.9	16.9	118	5.02	6.57	62.9	5.10	6.72	53.1
	F(O)	1.32	1.78	21.4	2.37	3.21	26.6	9.83	13.4	109	5.92	7.75	82.4	4.93	6.54	62.4
I (unfold-unfold)	E	2.75	3.22	11.1	3.76	4.71	21.0	10.5	17.5	305	11.0	14.2	53.1	7.05	9.89	104
	F	0.62	0.98	33.9	1.23	1.82	45.1	4.73	6.95	171	3.53	4.95	80.7	3.21	4.49	73.0
	F(H)	0.55	0.83	42.3	1.13	1.58	47.0	4.63	6.38	247	3.37	4.51	92.0	3.23	4.42	101
	F(C)	0.69	0.99	15.9	1.33	1.84	34.4	5.03	6.92	86.5	3.79	5.00	44.4	3.36	4.43	37.5
	F(N)	0.78	1.13	26.0	1.50	2.05	28.5	6.07	8.29	92.6	3.80	4.95	62.0	3.60	4.74	56.0
	F(O)	0.73	1.04	21.0	1.38	1.88	25.4	4.28	5.96	65.8	4.39	5.70	42.0	3.46	4.55	50.5
I (unfold-fold)	E	11.5	15.0	62.5	10.8	13.1	56.6	294	392	1770	11.0	14.2	53.1	50.8	71.2	418
	F	1.55	2.84	97.4	2.17	3.35	83.7	17.0	25.6	348	4.78	7.12	373	6.55	10.1	262
	F(H)	1.05	1.85	136	1.75	2.77	117	15.8	24.7	484	4.29	6.55	520	6.34	10.3	365
	F(C)	1.73	2.92	42.2	2.40	3.33	57.7	17.6	24.2	220	4.86	6.61	154	6.21	8.61	128
	F(N)	2.26	3.23	74.9	2.90	3.86	43.6	22.1	29.4	184	5.92	7.94	172	9.14	12.6	117
	F(O)	2.52	3.93	51.7	2.93	4.05	77.8	19.7	28.3	234	6.97	9.54	134	8.11	11.6	220
II (com)	E	1.51	1.95	21.7	3.51	4.37	18.3	26.8	36.8	192	55.9	69.9	305	17.2	21.8	103
	F	0.43	0.65	125	1.15	1.74	333	5.28	7.80	205	18.7	25.8	1400	5.93	8.26	456
	F(H)	0.38	0.56	176	1.04	1.51	302	4.93	6.97	290	18.2	24.7	1200	5.60	7.56	643
	F(C)	0.47	0.65	29.6	1.24	1.74	48.6	5.64	7.75	107	20.9	27.0	317	6.57	8.59	239
	F(N)	0.53	0.72	69.8	1.42	1.96	257	7.02	9.57	136	18.3	23.5	680	6.26	8.05	171
	F(O)	0.55	0.77	85.6	1.40	1.94	164	5.18	7.32	142	19.0	24.8	1430	6.52	8.56	136
II (incom-incom)	E	1.60	2.24	90.7	2.84	3.67	63.1	34.7	49.5	267	60.7	75.5	265	17.0	21.5	105
	F	0.39	0.63	309	1.10	1.68	324	4.63	7.01	205	19.1	26.3	1590	5.91	8.23	467
	F(H)	0.35	0.64	434	1.00	1.52	456	4.32	6.22	288	18.5	25.1	1787	5.57	7.52	656
	F(C)	0.41	0.58	51.5	1.18	1.66	108	4.96	6.98	93.2	21.7	27.8	305	6.52	8.51	249
	F(N)	0.47	0.70	100	1.31	1.86	152	6.15	8.60	92.4	18.6	24.2	1230	6.26	8.06	173
	F(O)	0.48	0.72	136	1.33	1.92	307	4.60	6.69	191	18.6	24.0	365	6.61	8.72	414
II (incom-unkn)	E	1.57	2.01	9.28	3.28	4.17	22.6	218	256	1240	57.8	72.7	294	19.1	24.3	125
	F	0.51	0.78	58.8	1.34	2.07	127	9.62	13.5	364	19.0	26.1	7790	6.07	8.50	192
	F(H)	0.42	0.63	55.2	1.12	1.72	180	10.1	14.7	517	18.5	25.1	832	5.73	7.80	216
	F(C)	0.57	0.79	22.0	1.49	2.11	51.1	9.48	12.5	120	21.5	27.6	310	6.68	8.79	123
	F(N)	0.65	0.87	26.4	1.69	2.28	60.7	11.9	15.4	129	18.5	23.8	598	6.52	8.40	135
	F(O)	0.68	0.95	59.0	1.69	2.35	91.0	9.87	13.2	149	18.0	23.2	294	6.66	8.79	193
III	E	0.90	1.14	4.68	1.52	1.91	11.6	1.70	2.13	9.85	4.84	5.90	18.1	1360	1360	1410
	F	0.16	0.25	14.3	1.88	3.13	148	0.76	1.26	41.2	1.90	2.55	233	4.85	6.42	639
	F(H)	0.30	0.42	10.8	1.36	1.94	41.9	0.86	1.26	13.4	2.13	3.04	120	6.26	8.29	650
	F(C)	0.15	0.23	14.1	1.92	3.17	146	0.74	1.24	38.2	1.88	2.51	231	4.78	6.30	632
	F(N)	0.39	0.57	8.02	1.55	2.18	38.9	1.31	2.04	35.4	2.68	3.56	60.9	6.41	8.45	262
	F(mol)	3.25	4.35	36.9	15.6	20.8	134	4.42	6.59	71.4	42.2	70.7	531	108	165	1107
IV	E	3.04	3.93	13.1	4.59	5.74	18.7	-	-	-	10.1	12.7	42.6	693	718	1300
	F	1.08	1.48	31.2	1.29	1.82	25.4	-	-	-	2.76	3.79	46.1	18.2	23.5	443
	F(H)	0.85	1.13	33.4	0.92	1.20	17.1	-	-	-	2.02	2.65	49.4	19.1	24.6	474
	F(C)	0.68	0.89	15.3	0.73	0.93	14.9	-	-	-	1.91	2.42	26.1	9.83	12.9	257
	F(N)	0.76	0.99	9.09	0.81	1.04	6.42	-	-	-	1.93	2.45	18.2	10.6	13.7	224
	F(Pb)	3.77	4.84	43.3	5.51	6.99	34.7	-	-	-	11.2	14.1	89.9	45.9	58.0	462
	F(I)	3.61	4.75	65.9	4.72	6.23	46.3	-	-	-	9.11	11.8	90.2	50.6	64.2	610
	F(mol)	4.82	6.24	79.3	4.76	6.04	49.0	-	-	-	11.7	14.8	110	133	169	1126

Table SI 8: MAE, RMSE, and MAX errors for relative energy and forces are reported in %, w.r.t. the standard deviations of the energies and forces in the reference DFT datasets.

References

[1] Ilyes Batatia, David P Kovacs, Gregor Simm, Christoph Ortner, and Gábor Csányi. MACE: Higher order equivariant message passing neural networks for fast and accurate force fields. *Adv. Neura.l Inf. Process. Syst.*, 35:11423–11436, 2022.

[2] Ilyes Batatia, Simon Batzner, David Peter Kovacs, Albert Musaelian, Gregor N. C. Simm, Ralf Drautz, Christoph Ortner, Boris Kozinsky, and Gabor Csanyi. The design space of E(3)-equivariant atom-centered interatomic potentials, 2022.

[3] David Peter Kovacs, Ilyes Batatia, Eszter Sara Arany, and Gabor Csanyi. Evaluation of the MACE force field architecture: From medicinal chemistry to materials science. *J. Chem. Phys.*, 159(4):044118, 07 2023.

[4] J. T. Frank, O. T. Unke, and K.-R. Müller. So3krates: Equivariant attention for interactions on arbitrary length-scales in molecular systems. *Adv. Neural Inf. Process Syst.*, 35:29400–29413, 2022.

[5] J. T. Frank, O. T. Unke, K.-R. Müller, and S. Chmiela. A Euclidean transformer for fast and stable machine learned force fields. *Nat. Commun.*, 15(1):6539, 2024.

[6] S. Chmiela, A. Tkatchenko, H. E. Sauceda, I. Poltavsky, K. T. Schütt, and K.-R. Müller. Machine learning of accurate energy-conserving molecular force fields. *Sci. Adv.*, 3(5):e1603015, 2017.

[7] S. Chmiela, H. E. Sauceda, K.-R. Müller, and A. Tkatchenko. Towards exact molecular dynamics simulations with machine-learned force fields. *Nat. Commun.*, 9(1):3887, 2018.

[8] S. Chmiela, V. Vassilev-Galindo, O. T. Unke, A. Kabylda, H. E. Sauceda, A. Tkatchenko, and K.-R. Müller. Accurate global machine learning force fields for molecules with hundreds of atoms. *Sci. Adv.*, 9(2):eadf0873, 2023.

[9] S. Chmiela, H. E Sauceda, Igor Poltavsky, and A. Müller, K.-R.and Tkatchenko. sGDML: Constructing accurate and data efficient molecular force fields using machine learning. *Computer Physics Communications*, 240:38–45, 2019.

[10] C. E. Rasmussen and C. K.I. Williams. *Gaussian processes for machine learning*, volume 1. Springer, 2006.

[11] A. P. Bartók and G. Csányi. Gaussian approximation potentials: A brief tutorial introduction. *Int. J. Quantum Chem.*, 115(16):1051–1057, 2015.

[12] Volker L Deringer, A. P. Bartók, Noam Bernstein, David M Wilkins, Michele Ceriotti, and Gábor Csányi. Gaussian process regression for materials and molecules. *Chem. Rev.*, 121(16):10073–10141, 2021.

[13] E. Snelson and Z. Ghahramani. Local and global sparse Gaussian process approximations. In *Artificial Intelligence and Statistics*, pages 524–531. PMLR, 2007.

[14] A. S. Christensen, F. A. Faber, B. Huang, L. A. Bratholm, A. Tkatchenko, K.-R. Müller, and A. O. von Lilienfeld. Qml: A python toolkit for quantum machine learning. *preprint*, 2017.

[15] Nicholas J. Browning, Felix A. Faber, and O. Anatole von Lilienfeld. GPU-accelerated approximate kernel method for quantum machine learning. *J. Chem. Phys.*, 157(21):214801, 12 2022.

[16] A. Rahimi and B. Recht. Random features for large-scale kernel machines. *Adv. Neural Inf. Process Syst.*, 20, 2007.

[17] F. X. X. Yu, A. T. Suresh, K. M. Choromanski, D. N. Holtmann-Rice, and S. Kumar. Orthogonal random features. *Adv. Neural Inf. Process Syst.*, 29, 2016.

[18] A. S. Christensen, L. A. Bratholm, F. A. Faber, and O. A. von Lilienfeld. FCHL revisited: Faster and more accurate quantum machine learning. *J. Chem. Phys.*, 152(4), 2020.

[19] A. P. Bartók, M. C. Payne, R. Kondor, and G. Csányi. Gaussian approximation potentials: The accuracy of quantum mechanics, without the electrons. *Phys. Rev. Lett.*, 104(13):136403, 2010.

[20] A. P. Bartók, R. Kondor, and G. Csányi. On representing chemical environments. *Phys. Rev. B*, 87(18):184115, 2013.

[21] V. L. Deringer, M. A. Caro, and G. Csányi. Machine learning interatomic potentials as emerging tools for materials science. *Adv. Mater.*, 31(46):1902765, 2019.

[22] Volker L. Deringer and Gábor Csányi. Machine learning based interatomic potential for amorphous carbon. *Phys. Rev. B*, 95:094203, 2017.

[23] Bingqing Cheng, Ryan-Rhys Griffiths, Simon Wengert, Christian Kunkel, Tamas Stenczel, Bonan Zhu, Volker L. Deringer, Noam Bernstein, Johannes T. Margraf, Karsten Reuter, and Gabor Csanyi. Mapping materials and molecules. *Acc. Chem. Res.*, 53(9):1981–1991, 2020.

[24] Elena Gelžinytė, Simon Wengert, Tamás K. Stenczel, Hendrik H. Heenen, Karsten Reuter, Gábor Csányi, and Noam Bernstein. wfl Python toolkit for creating machine learning interatomic potentials and related atomistic simulation workflows. *J. Chem. Phys.*, 159(12):124801, 09 2023.

[25] Justin Gilmer, Samuel S Schoenholz, Patrick F Riley, Oriol Vinyals, and George E Dahl. Neural message passing for quantum chemistry. In *International conference on machine learning*, pages 1263–1272. PMLR, 2017.

[26] Oliver T Unke and Markus Meuwly. PhysNet: A neural network for predicting energies, forces, dipole moments, and partial charges. *J. Chem. Theory Comput.*, 15(6):3678–3693, 2019.

[27] Atılım Günes Baydin, Barak A Pearlmutter, Alexey Andreyevich Radul, and Jeffrey Mark Siskind. Automatic differentiation in machine learning: a survey. *J. Mach. Learn. Res.*, 18(1):5595–5637, 2017.

[28] Martín Abadi, Paul Barham, Jianmin Chen, Zhifeng Chen, Andy Davis, Jeffrey Dean, Matthieu Devin, Sanjay Ghemawat, Geoffrey Irving, Michael Isard, Manjunath Kudlur, Josh Levenberg, Rajat Monga, Sherry Moore, Derek G. Murray, Benoit Steiner, Paul Tucker, Vijay Vasudevan, Pete Warden, Martin Wicke, Yuan Yu, and Xiaoqiang Zheng. Tensorflow: A system for large-scale machine learning. In *12th USENIX symposium on operating systems Design and Implementation (OSDI 16)*, pages 265–283, 2016.

[29] Sashank J Reddi, Satyen Kale, and Sanjiv Kumar. On the convergence of adam and beyond. *arXiv preprint arXiv:1904.09237*, 2019.

[30] Ask Hjorth Larsen, Jens Jørgen Mortensen, Jakob Blomqvist, Ivano E Castelli, Rune Christensen, Marcin Dułak, Jesper Friis, Michael N Groves, Bjørk Hammer, Cory Hargus, Eric D Hermes, Paul C Jennings, Peter Bjerre Jensen, James Kermode, John R Kitchin, Esben Leonhard Kolsbjerg, Joseph Kubal, Kristen Kaasbjerg, Steen Lysgaard, Jón Bergmann Maronsson, Tristan Maxson, Thomas Olsen, Lars Pastewka, Andrew Peterson, Carsten Rostgaard, Jakob Schiøtz, Ole Schütt, Mikkel Strange, Kristian S Thygesen, Tejs Vegge, Lasse Vilhelmsen, Michael Walter, Zhenhua Zeng, and Karsten W Jacobsen. The atomic simulation environment – a Python library for working with atoms. *J. Phys. Condens. Matter*, 29(27):273002, 2017.

[31] Thomas Bischoff, Bastian Jäckl, and Matthias Rupp. Hydrogen under pressure as a benchmark for machine-learning interatomic potentials. *arXiv preprint arXiv:2409.13390*, 2024.

[32] Stephen R. Xie, Matthias Rupp, and Richard G. Hennig. Ultra-fast interpretable machine-learning potentials. *npj Comput. Mater.*, 9:162, 2023.
